# Analysis strategies for time-resolved X-ray solution scattering at high repetition rate XFEL sources

**DOI:** 10.1107/S1600577525009737

**Published:** 2026-01-01

**Authors:** Morten Lunn Haubro, Diana Bregenholt Zederkof, Dmitry Khakhulin, Hazem Yousef, Yifeng Jiang, Ivette Jazmin Bermudez Macias, Thomas Kluyver, Frederico Lima, Mykola Biednov, Christopher Milne, Yohei Uemura, Peter Zalden, Hao Wang, Asmus Ougaard Dohn, Kerstin Mitterer, Verena Markmann, Oliver Ohlson, Martha Tordis Wachter-Lehn, Philipp Lenzen, Benedikte Knorr Jensen, Alberte Høholdt, Tim Brandt van Driel, Amke Nimmrich, Elli Selenius, Gianluca Levi, Klaus Braagaard Møller, Martin Meedom Nielsen, Kristoffer Haldrup

**Affiliations:** a Department of Physics Technical University of Denmark 2800Kongens Lyngby Denmark; b European XFEL Holzkoppel 4 22869Schenefeld Germany; c Department of Chemistry Technical University of Denmark 2800Kongens Lyngby Denmark; d Linac Coherent Light Source SLAC National Accelerator Laboratory Menlo Park CA94025 USA; e Department of Chemistry and Molecular Biology University of Gothenburg 40530Gothenburg Sweden; f Department of Chemistry University of Washington Seattle WA98195 USA; g Science Institute and Faculty of Physical Sciences University of Iceland Reykjavík Iceland; h Department of Chemical and Pharmaceutical Sciences University of Trieste 34127Trieste Italy; Paul Scherrer Institut, Switzerland

**Keywords:** XFEL, high repetition rate, sample destruction, solution scattering

## Abstract

With the start of the user program at the European XFEL in 2017, and more recently with the LCLS-II upgrade, the X-ray repetition rate at X-ray free-electron lasers (XFELs) has been pushed into the kilo- and megahertz regimes. These high X-ray repetition rates provide an increase in the integrated flux at these facilities by orders of magnitude, potentially facilitating measurements that were previously infeasible due to signal-to-noise constraints. However, the high repetition rates lead to new challenges for sample delivery and a shorter time for the sample to recover between X-ray pulses. For solution-phase techniques, the X-ray–sample interactions will strongly perturb or even vaporize the sample jet. Although the sample can be replenished, up to X-ray repetition rates of ∼100 kHz, by flowing the jet at high speeds, this does not completely mitigate the jet perturbations. In this work, we present a characterization of the jet perturbations induced by the high X-ray repetition rates at the European XFEL. We show how these can introduce background signals in time-resolved X-ray solution scattering data measured at the Femtosecond X-ray Experiments (FXE) instrument. We show that it is possible to mitigate these experimental artifacts by employing an alternating excitation scheme combined with careful background subtraction and that implementing this approach in the experimental design outperforms more simple background subtraction schemes. The methodology, the observations and analysis results are discussed in relation to the evolving landscape of XFEL sources.

## Abstract 2

An analysis of experimental artifacts in time-resolved X-ray solution scattering data acquired at high repetition rate XFEL sources, specifically at the FXE instrument at the European XFEL, is presented.

## 1. Introduction

**Figure 1:**
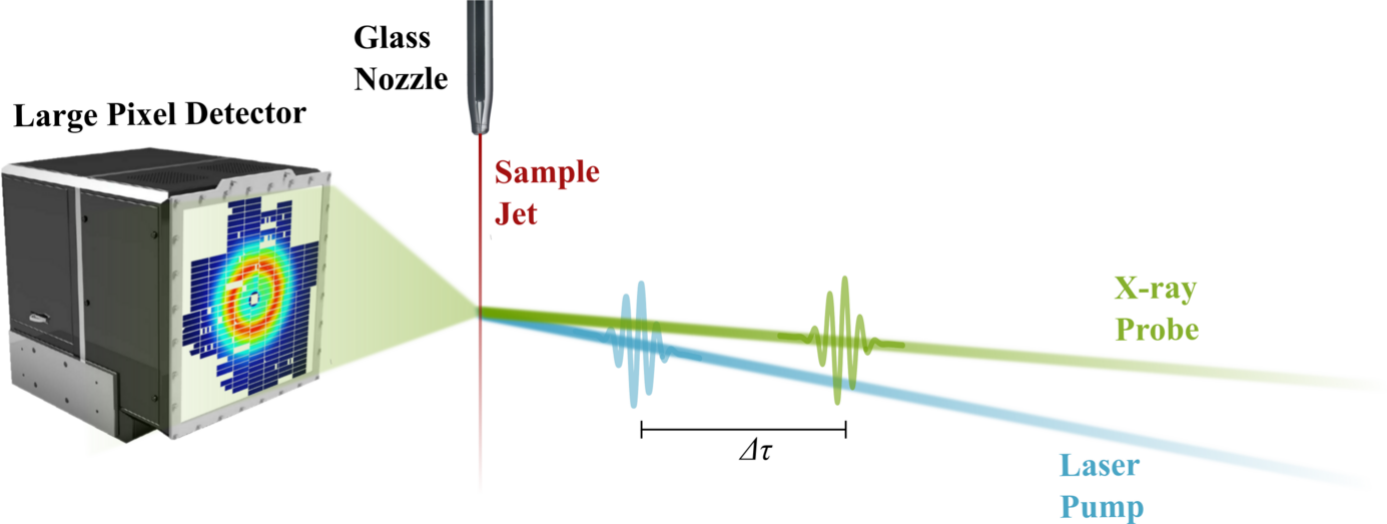
Schematic of the typical setup utilized for TR-XSS experiments at FXE. The sample solution is delivered in a 30–100 µm liquid jet under high pressure. An optical laser pump pulse promotes the photoactive sample to an excited electronic state and the subsequent atomic structural evolution is probed by an X-ray pulse arriving a time delay Δτ after photoexcitation. The scattered X-ray light is detected by the 2D LPD detector.

Time-resolved X-ray solution scattering (TR-XSS) as performed at X-ray free electron lasers (XFELs) has emerged as a powerful tool for probing atomic-scale structural dynamics on a femto- to microsecond time scale (Gaffney, 2021 ▸; Choi *et al.*, 2022 ▸). TR-XSS utilizes an optical laser pump, X-ray probe setup, thereby exciting a photoactive solute and probing the structural changes associated with the excited electronic configurations. A schematic of a typical TR-XSS setup is presented in Fig. 1 ▸.

The structural sensitivity of X-ray scattering (Jeong *et al.*, 2022 ▸) combined with the high time resolution provided by the ∼30 femtosecond (fs) X-ray pulses produced at XFELs makes TR-XSS a powerful tool for probing ultrafast atomic-scale dynamics. In a typical TR-XSS experiment, the sample system is delivered in a liquid jet and excited with a fs optical laser pulse. Subsequently, the sample is probed with an X-ray pulse at a time delay Δτ after photoexcitation. The time-dependent scattering signals from the photoexcited system, *S*_on_(Δτ), are measured with a 2D area detector. As the scattering response associated with structural reorganization is quite weak compared with the static cross section, a *difference* scattering signal, Δ*S*(Δτ), is calculated to isolate the effects of the laser excitation, 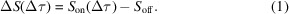
*S*_off_ denotes a scattering signal measured without prior laser excitation – corresponding to scattering from the system in the ground state. TR-XSS has been leveraged to observe, for example, bond formation (van Driel *et al.*, 2016 ▸) and dissociation events (Nimmrich *et al.*, 2023 ▸; Choi *et al.*, 2021 ▸), wave packet dynamics (Haldrup *et al.*, 2019 ▸; Kunnus *et al.*, 2020 ▸) and solvent dynamics (Biasin *et al.*, 2021 ▸; Ki *et al.*, 2021 ▸; Markmann *et al.*, 2024 ▸; Katayama *et al.*, 2023 ▸).

So far, most studies have been conducted on strong-scatter sample systems containing at least one atom with a high atomic number, *Z*, in order to maximize the strength of the solute-specific scattering signal. However, many functional or bio-relevant photo-active molecules consist of mainly low-*Z* atomic species. The prospect of studying such compounds with TR-XSS is becoming more feasible with the advent of high-repetition-rate XFELs, such as the European XFEL and Linac Coherent Light Source (LCLS)-II-HE. The significantly higher integrated flux of these MHz facilities potentially increases the signal-to-noise ratio by several orders of magnitude to what can be achieved at the more common ∼100 Hz facilities. Although TR-XSS measurements typically use a fast-flowing jet (order of ∼10 m s^−1^) ensuring sample renewal between pulses, the higher repetition rates (kHz–MHz) can dramatically increase the magnitude of sample perturbation observed in the measured scattering signals as the time for the absorbed X-ray energy to be dissipated in the sample and for the sample to be replenished between successive scattering events is very short. For example, with an X-ray photon energy of 9.3 keV, a 100 µm-thick water jet absorbs roughly 6% of the incident intensity. For a pulse energy of 300 µJ and assuming a cylindrical beam with a diameter of 20 µm, this leads to a temperature increase in the probed region of 140 K (see Section S1 of the supporting information for details). It should be noted that the magnitude of the temperature increase depends sensitively on the X-ray energy, X-ray spot size, and the thermodynamic properties and the X-ray attenuation coefficient of the solvent. While the specific solvent is typically integral to the experiment, there can be flexibility in terms of the X-ray energy and for instance increasing the X-ray photon energy from, for example, 9 keV to 18 keV would reduce this temperature increase to ∼20 K as the absorption depends on energy as *E*^−3^ (see also Fig. S1 in the supporting information).

In previous work, Stan *et al.* (2016 ▸) studied the interaction between a liquid jet and an XFEL beam at the LCLS and found that interaction with SASE X-ray pulses triggered explosion of the water jet on a ∼200 ns time scale. Following the explosion, shock waves were observed to propagate away from the area of the explosion, perturbing the otherwise intact regions of the jet. Similar effects have since been observed with X-ray imaging following explosions induced by visible light (Hagemann *et al.*, 2021 ▸). Considering the European XFEL, X-rays are produced in pulse trains with an intra-train temporal spacing of the X-ray pulses reaching down to 220 ns (Decking *et al.*, 2020 ▸; Galler *et al.*, 2019 ▸). At this 4.5 MHz repetition rate, with a jet flow speed similar to what is typically used at ∼100 Hz XFELs (∼10 m s^−1^) and with a typical X-ray beam size of 10 µm, already by the second pulse in each train, the jet has been exploded, and a strongly perturbed scattering pattern is obtained. By increasing the flow speed of the sample jet (Khakhulin *et al.*, 2020 ▸; Lima *et al.*, 2023 ▸; Biednov *et al.*, 2023 ▸) to ∼60 m s^−1^, it is possible to operate at several hundred kHz while fully replenishing the sample between pulses, ensuring that each measurement probes the same photo-physical properties as the previous. However, the hydrodynamic perturbations travel through the liquid sample at or even above the speed of sound (Stan *et al.*, 2016 ▸), making it impossible to flow the sample fast enough that the pressure and temperature of the interaction volume remain constant between pulses, unless the interaction volume becomes separated by a distance much greater than the attenuation length of the perturbations.

In addition to the challenges stemming from sample perturbation and destruction, high-repetition X-ray experiments also put additional requirements on the detection systems. At 4.5 MHz, the 220 ns interval between scattering events is not enough to fully read out an image and clear the charge from a large area (≥1 Mpix) 2D detector. To solve this issue, detectors such as the AGIPD (Adaptive Gain Integrating Pixel Detector) (Mezza *et al.*, 2016 ▸) and the LPD (Large Pixel Detector) (Veale *et al.*, 2017 ▸) have been developed for the European XFEL. Their on-chip storage that is read out between trains facilitates operation at MHz intra-train repetition rate. However, as a result of the on-chip storage, each scattering pattern needs to be corrected with appropriate offset (‘pedestal’) and gain for each pixel, gain stage and memory cell (Wheater *et al.*, 2022 ▸).

In this work, we provide an overview of some of the challenges associated with high-repetition-rate experiments as they arise in a series of TR-XSS experiments conducted at the FXE Instrument at the European XFEL (Galler *et al.*, 2019 ▸; Khakhulin *et al.*, 2020 ▸). The observed changes in the acquired scattering patterns are interpreted in terms of the underlying physics, and their dependence on pulse intensity as well as on repetition rate is characterized. In combination with model-independent analysis, this makes it possible to identify also detector-related contributions to the changes in the acquired scattering data and mitigation strategies are proposed and discussed.

## 2. Experimental details

The analysis presented here evaluates data sets from three independent scientific campaigns at the FXE instrument. For ease of reference, these experiments are labeled A, B and C. The experiments were carried out in 2022 (A, B) and 2024 (C). For experiment A, the sample was [Fe(bpy)(CN_4_)]^2−^ (bpy = 2,2′-bipyridine), which upon photoexcitation into states of mainly metal-to-ligand charge transfer (MLCT) character decays into states of mainly triplet metal centered (^3^MC) character (Kjaer *et al.*, 2018 ▸; Ledbetter *et al.*, 2020 ▸; Zederkof *et al.*, 2022 ▸; Jay *et al.*, 2019 ▸; Polonius *et al.*, 2025 ▸). The MLCT states are expected to be structurally similar to the ground state, while the MC states are expected to be associated with an expansion of the Fe ligand bond lengths (Zederkof *et al.*, 2022 ▸; Polonius *et al.*, 2025 ▸). During experiments B and C, the sample system was a small organic molecular system: a hemithioindigo compound where the stilbene fragment is a 3,5-dimethyljulolidine group [indicated as ‘*Z* − 2’ by Wiedbrauk *et al.* (2016 ▸)] in experiment B and TIPSpn [bis(triisopropylsilylethynyl) pentacene] (Walker *et al.*, 2013 ▸) in experiment C. The difference scattering signal associated with the photoinduced structural changes of these organic compounds is small, and thus the dominant signal will be that of the solvent heating upon energy dissipation of the photoexcited solute (Kjaer *et al.*, 2013 ▸). Key experimental parameters of all three experiments are detailed in Table 1 ▸. The setup used, briefly described in the following, is similar to what has been previously reported (Khakhulin *et al.*, 2020 ▸; Lima *et al.*, 2023 ▸). For all the experiments, X-rays were generated at an intra-train repetition rate of 141 kHz, resulting in ∼50 pulses per train with a temporal spacing of 7 µs, at energies of 9.3, 12.0 and 9.1 keV during the three experiments, respectively, but for consistency of presentation and ease of comparison all data plots use the same *q*-range, *q* = 0.4 Å^−1^ to 4 Å^−1^. For all three experiments the ∼50 fs X-ray pulses were focused to a FWHM of ∼30 µm at the interaction point, while the ∼70 fs laser pulses were focused to a FWHM of ∼60 µm. The sample was delivered as a round liquid jet with a 100 µm diameter at a flow speed of ∼60 m s^−1^, ensuring that the interaction volume was replenished between pulses.

X-ray scattering was recorded using the LPD detector system, designed for the specific needs of diffuse scattering and diffraction experiments at high X-ray repetition rates (Veale *et al.*, 2017 ▸). The LPD employs 512 memory cells in each pixel, each of which can store the measured scattering from a single X-ray probe pulse, data which is then read out between trains. The LPD offers three different gain stages (high, medium and low), with the option for both auto-switching and simultaneous readout (Wheater *et al.*, 2022 ▸). The gain modes used in these experiments are noted in Table 1 ▸.

### 2.1. Excitation scheme

**Figure 2:**
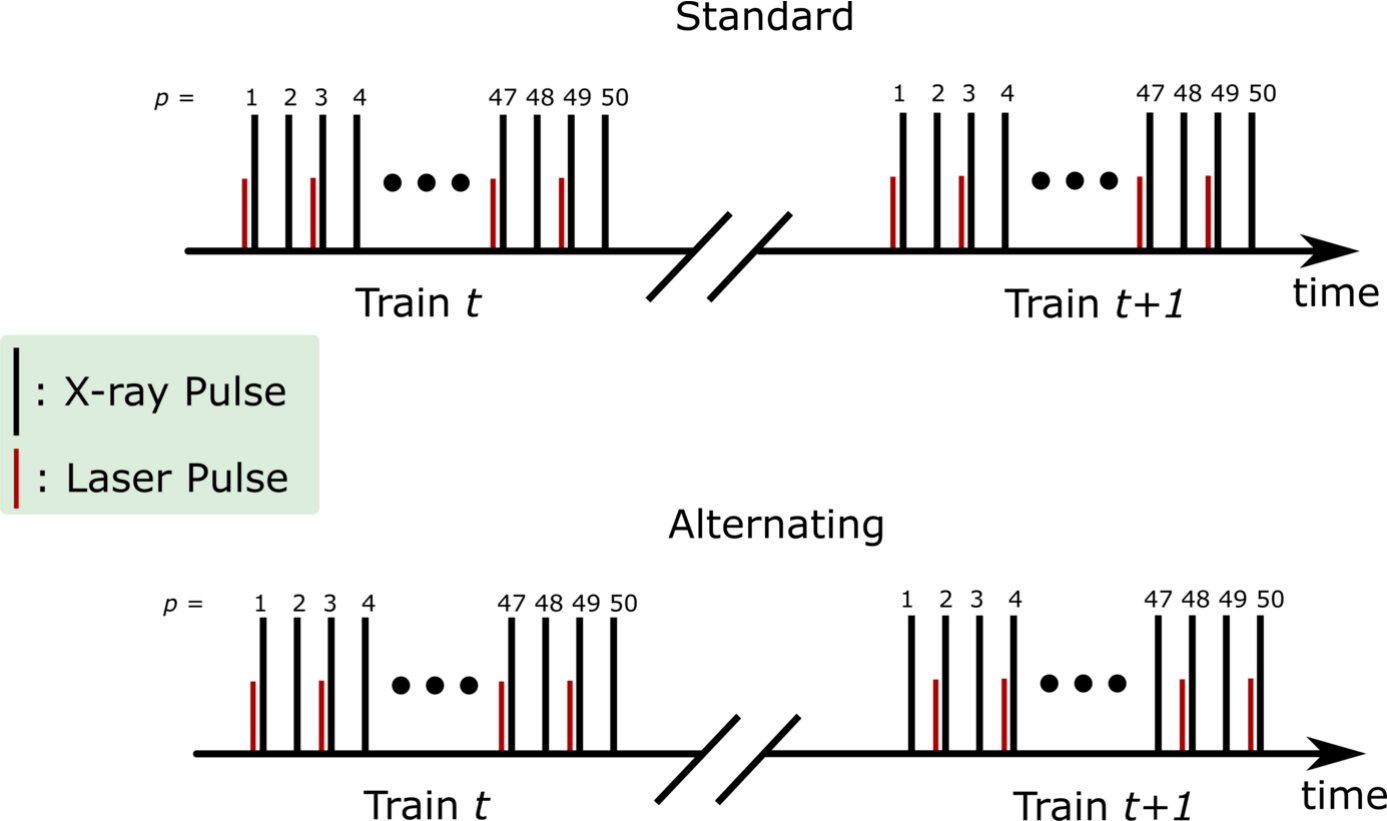
The two different excitation schemes investigated in this work. The *standard* scheme, where every second measurement is laser on, starting with the first in each train, and the *alternating* scheme, where every second measurement is ‘laser on’, alternating between starting with the first and second pulse of each train.

In the following, a *measurement* will be designated as the recording of scattering from a single probe pulse. In order to minimize shot-to-shot variations, every second measurement at FXE is a ‘laser off’ measurement. This makes it possible to construct the difference scattering signal [equation (1)] from consecutively measured scattering patterns, minimizing the effects of systematic drifts in X-ray intensity and background from air scattering. In this work, we present an investigation of two different excitation schemes. First, the *standard* excitation scheme, presented in the upper panel of Fig. 2 ▸, where the excitation pattern for each train is the same, *i.e.* the first measurement in each train is an ‘on’ measurement, the second is ‘off’, and so on. Alternatively, the *alternating* excitation scheme, presented in the lower panel of Fig. 2 ▸, can be used. As with the standard excitation scheme, every second measurement is ‘laser off’. However, in contrast to the standard scheme, where the excitation pattern is identical for all trains, the order of the ‘laser on’ and ‘laser off’ measurements alternates from train to train. In other words, if the first train is on–off–on–off, the second train will be off–on–off–on. In the case where the sample jet is perturbed by both the X-rays and the visible laser and that there are systematic differences between various memory cells, the choice of excitation pattern may influence the shape of the difference scattering signal. The different merits of the two excitation schemes are discussed in Section 7.

### 2.2. Data reduction

**Figure 3:**
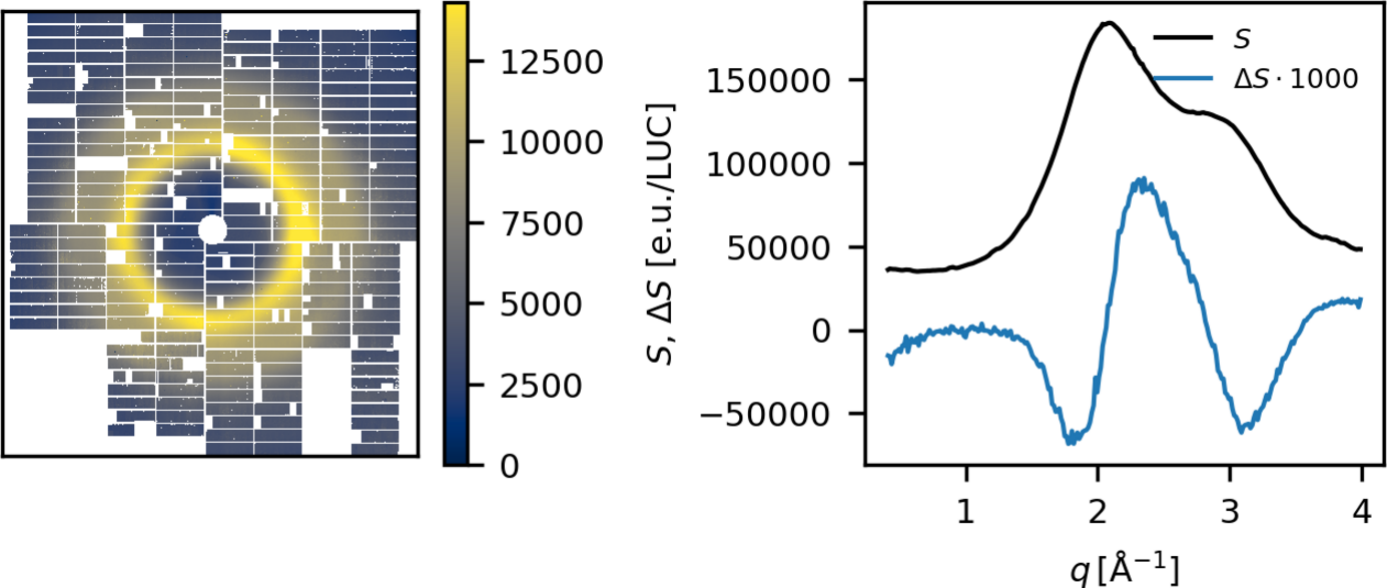
Left: an example of a detector image showing the scattering pattern measured during experiment A, where a mask and solid angle and polarization correction have been applied. Right: an example of the azimuthally integrated scattering signal *S* and difference scattering signal Δ*S* multiplied by a factor of 1000 for clarity, both plotted as a function of momentum transfer *q*.

After each train, LPD raw images are stored in the XFEL database for further post-processing. The detector Pedestal (measured under no X-ray exposure) is subtracted from each image. Solid angle (Bösecke & Diat, 1997 ▸) and polarization corrections (Hura *et al.*, 2000 ▸) are then applied to the corrected detector images, and individual *bad* pixels are removed with a mask. The left panel of Fig. 3 ▸ shows an example of a corrected scattering pattern.

Subsequently, the detector images are azimuthally integrated using the *PyFAI* software package (Ashiotis *et al.*, 2015 ▸), yielding scattering curves as a function of momentum transfer, defined as *q* = 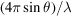, where λ is the X-ray wavelength and 2θ the scattering angle. Fig. 3 ▸, right panel, presents an example of the azimuthally integrated scattering signal *S* and the difference signal Δ*S* from experiment A, calculated as described in the following. For the most recent experiment (C), the integration was implemented in the *DAMNIT* tool (https://damnit.readthedocs.io/) developed by the European XFEL Data Analysis group. Each of these azimuthal curves *S*(*q*) is then self-normalized (in the following, the superscript ‘n’ denotes self-normalization) according to 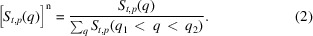
For all the data presented here, *q*_1_ = 0.7 Å^−1^, *q*_2_ = 4 Å^−1^. The subscripts *t* and *p* denote the train and pulse number, respectively, uniquely identifying each scattering pattern. This normalization minimizes the variations in the scattering intensity as a result of fluctuations in the incident X-ray beam intensity. Subsequently, the difference scattering signal, Δ*S*(*q*, Δτ_*i*_), is constructed from all trains (*t* = 1…*N*_*t*_) and all pulses (*p* = 1…*N*_*p*_) measured with a given time delay Δτ_*i*_ according to (for the *standard* excitation scheme)
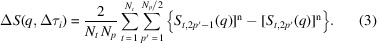
Here, the ‘laser on’ and ‘laser off’ measurements are denoted as the odd and even pulses in each train, respectively, assuming the *standard* excitation scheme. The data are then scaled to the smallest stochiometrically representative unit, known as the liquid unit cell (LUC), to convert the arbitrary intensity scale of the scattering pattern to electron units per liquid unit cell, allowing quantitative analysis (Haldrup *et al.*, 2010 ▸) (see also Section S2 of the supporting information including Fig. S2). An example of the resulting difference scattering signal is compared with the absolute scattering signal in the right panel of Fig. 3 ▸. Note that the difference scattering is scaled by a factor of 1000 to facilitate the comparison with the absolute scattering signal.

**Figure 4:**
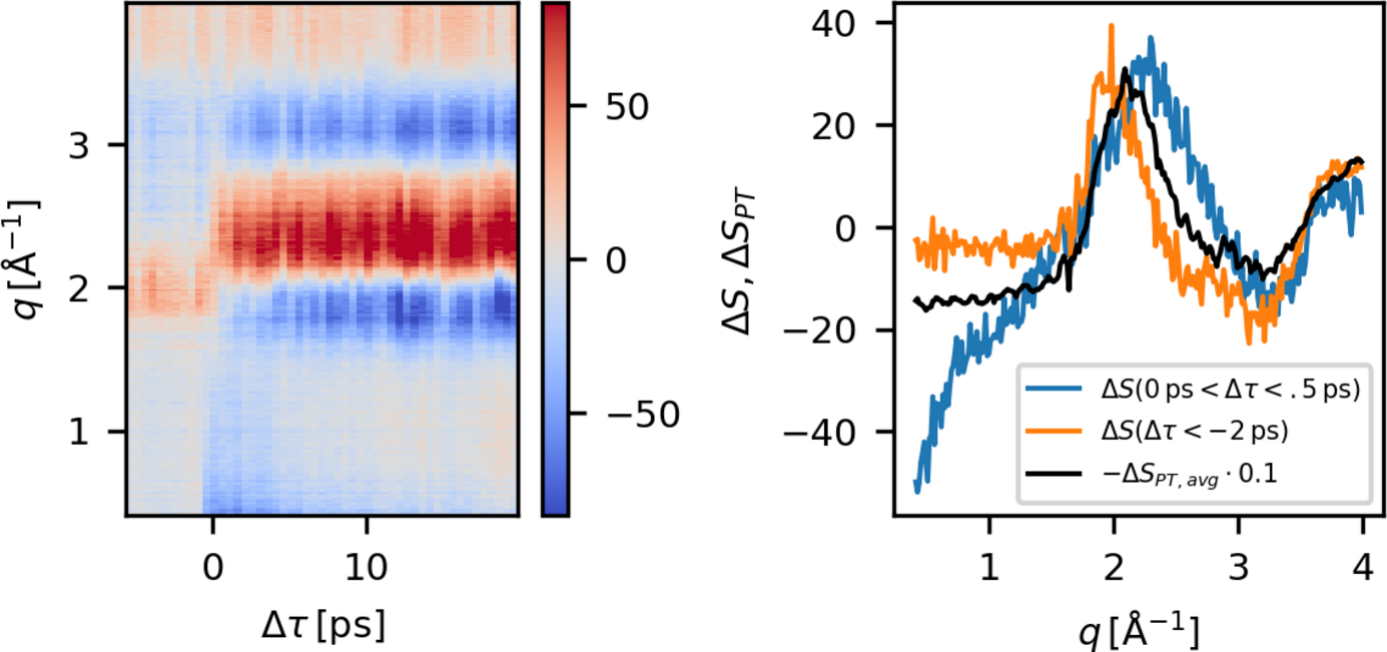
An example of the difference scattering signals upon preliminary data reduction. Left: Δ*S*(*q*, Δτ) as calculated according to equation (3). The data were measured during experiment A on the [Fe(bpy)(CN_4_)]^2−^ complex in water. Right: averaged difference signals of Δ*S*, before time zero (orange) and early delays (blue), as well as the average *pulse train difference* (black) calculated according to equation (4). These data were measured during experiment A.

Repeating this approach for each time delay yields the time-dependent, Δτ, difference scattering data, Δ*S*(*q*, Δτ_*i*_), as presented in the left panel of Fig. 4 ▸. The data presented are from experiment A and were measured with water as the solvent. There is a clear onset of signal around time zero, Δτ = 0, in the *q* = 1.5–4 Å^−1^ region, resembling the known difference scattering signal arising from water heating (Kjaer *et al.*, 2013 ▸). The negative signal present for *q* ≤ 1.5 Å^−1^ arises from Fe—N bond length elongation in accordance with the expected signals from computational work on this complex (Zederkof *et al.*, 2022 ▸; Polonius *et al.*, 2025 ▸) and also observed for similar Fe-centered compounds (Kjaer *et al.*, 2018 ▸).

For negative time delays, Δτ < 0, it is expected that Δ*S* = 0, but a significant background is present and is observed to be independent of the time delay Δτ. Fig. 4 ▸, right panel, shows a comparison between the averaged pump–probe signal for the first 0.5 ps after time zero, the background, Δ*S*_BG_, which is obtained as an average of Δ*S*(Δτ < −2 ps), and the average *pulse train difference*, Δ*S*_PT_, with the definition and utility of the latter presented in the next section. The background contribution, Δ*S*_BG_ (orange), is of similar magnitude as the actual pump–probe signal (blue), which potentially makes quantitative analysis more challenging. A proposed solution to this is to subtract a reference signal, measured at negative pump–probe delay, from the delay scans (Lima *et al.*, 2023 ▸). If, as Fig. 4 ▸ (left) seems to indicate, the background is constant, this method will recover the part of Δ*S*(*q*, Δτ) that arises from photoexcitation of the solute molecule. This approach is, however, sensitive to systematic drifts that may cause the magnitude and shape of the persistent background contribution (Δ*S*_BG_) to drift over the course of a delay scan.

**Figure 5:**
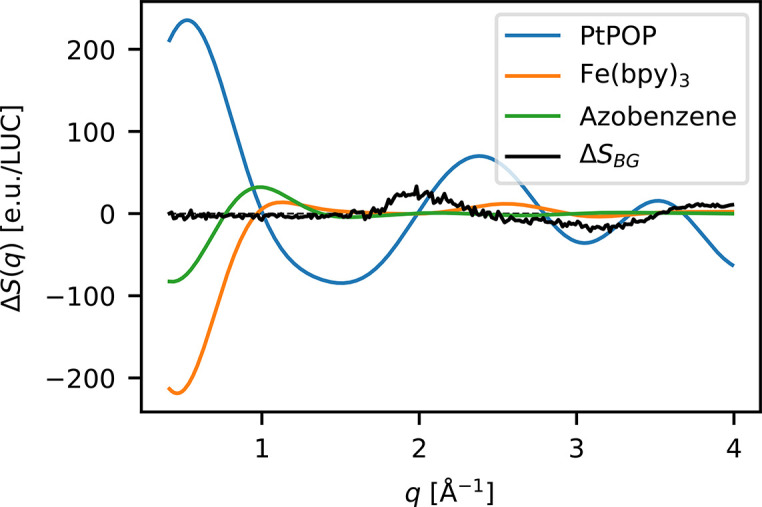
A comparison between the measured background from experiment A, Δ*S*_BG_, and simulated difference scattering signals arising from the high spin state of Fe(bpy)_3_, the triplet state of PtPOP, and the expected signal from a *cis*–*trans* isomerization of an azobenzene. All simulated signals are scaled to a 10% excitation fraction.

Fig. 5 ▸ shows a comparison between Δ*S*_BG_ identified in the data set from experiment A compared with simulated difference scattering signals arising from photoexcitation of a di-platinum complex [Pt_2_POP_4_ (Levi *et al.*, 2018 ▸)], an Fe-centered coordination complex [Fe(bpy)_3_ (Kjaer *et al.*, 2019 ▸)] and an azobenzene (Merritt *et al.*, 2021 ▸), all of which have been scaled to a reasonable 10% excitation fraction. In particular for *Q* > 1.5 Å^−1^ this comparison highlights how the presence of a structured background signal, like the one observed for experiment A, may complicate or bias quantitative analysis if not properly removed or accounted for, even for systems exhibiting very strong difference signals like the Pt_2_POP_4_ complex. Indeed, for structural dynamics in low-*Z* systems such as the *cis*–*trans* isomerization of the azobenzene presented in Fig. 5 ▸, the presence of artifacts of this magnitude may completely obscure the solute-related difference signals in the acquired data.

In the following, the origin of Δ*S*_BG_ is explored, and methods to characterize and minimize it are presented. Since the high repetition rates may cause sample perturbation during pulse trains, an approach is developed to characterize the changes to the scattering signal over the course of the train.

## 3. The pulse train difference

To isolate the pulse-dependent artifacts that are not related to any solute dynamics, the pulse train difference is defined as 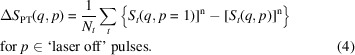
Note that only ‘laser off’ measurements are used to calculate Δ*S*_PT_ to avoid including the excited structural signal of the sample for this analysis of the pulse-dependent artifacts. In equation (4), *S* denotes the azimuthally integrated scattering pattern, *p* denotes the pulse number of the given scattering pattern, and *t* denotes the train index. As before [equation (2)], the superscript n denotes self-normalization. Thus, all off pulses within a given pulse-train are considered relative to the first pulse. By averaging the same pulse numbers pairwise for all *N*_*t*_ trains in a given data set, the random variations between individual trains are minimized. While the difference scattering signal, Δ*S*(*q*, Δτ_*i*_) [equation (3)], is designed to isolate the difference scattering pattern stemming from photoexcitation of the sample at a time delay Δτ, the pulse train difference [equation (4)] is designed to isolate possibly cumulative effects of all previous pulses in the train on the scattering signal from pulse *p*. These effects include X-ray and laser induced sample perturbations, but may also include detector artifacts, X-ray energy drifts, *etc*.

**Figure 6:**
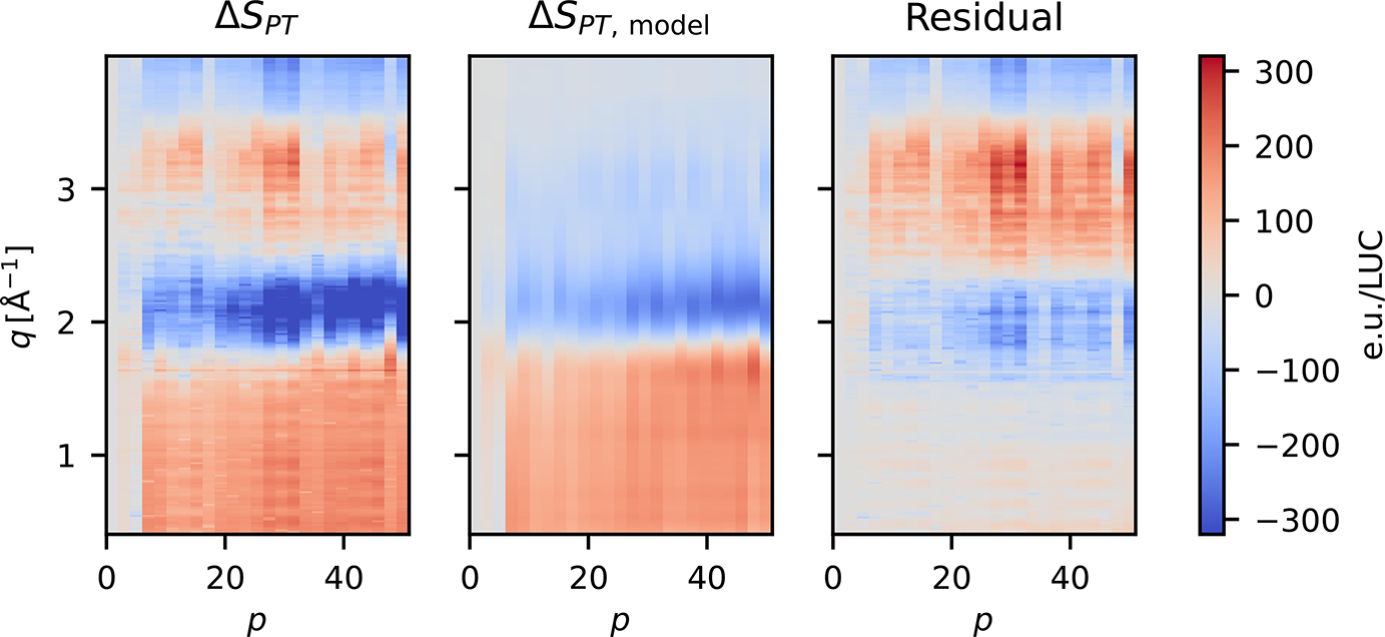
Left: the pulse train difference, Δ*S*_PT_(*q*, *p*), calculated from the laser off measurements of a pump–probe scan. Middle: the best fit to Δ*S*_PT_ of the model defined by equation (5). Right: the residual of the model fit. These data were measured during experiment A.

Fig. 4 ▸ (right) compares the averaged difference scattering at negative time delays, Δ*S*(*q*, Δτ < −2 ps) (orange), with the averaged difference scattering for early delays, Δ*S*(*q*, 0 < Δτ < 0.5 ps) (blue), and an average of the pulse-train difference, Δ*S*_PT, avg_ (black), for a single delay scan. Note that Δ*S*_PT, avg_ is scaled down by a factor of ten to be of comparable magnitude with Δ*S*. Comparison of the averaged background signal Δ*S*_BG_ (orange) and the averaged pulse-train difference Δ*S*_PT, avg_ (black) [Fig. 4 ▸ (right)] shows similar but not identical signals. The differences in both shape and magnitude are assigned to the fact that the background signal Δ*S*_BG_ is constructed from nearest neighbor scattering patterns, while the pulse-train differences Δ*S*_PT_ include differences between the first pulse and all the remaining ‘off’ pulses. The similarities suggest that the origin of the non-zero signals observed in the averaged pulse-train differences, Δ*S*_PT, avg_, is the same as the origin of the background, Δ*S*_BG_. The averaged pulse-train differences, Δ*S*_PT_, calculated for ∼15 min of data (∼9000 trains) is plotted in Fig. 6 ▸ (left). Here, the data have been scaled to the LUC of the 20 m*M* [Fe(bpy)(CN)_4_]^2−^ sample solution (2750 water molecules for each solute molecule).

**Figure 7:**
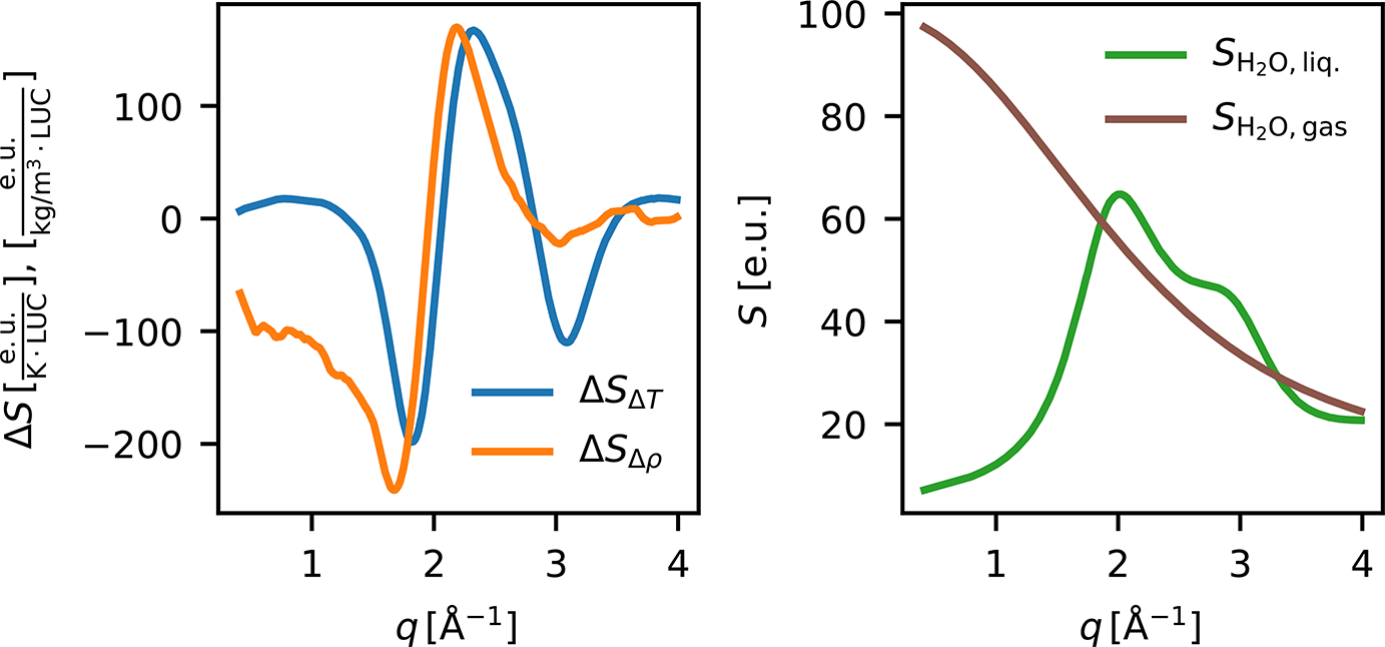
The scattering components contributing to the fitted model. Left: Δ*S*_Δ*T*_ and Δ*S*_Δρ_ from Kjaer *et al.* (2013 ▸), scaled by the number of water molecules in the LUC (2750). Right: 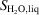, the scattering signal from liquid water scaled to one water molecule (Skinner *et al.*, 2013 ▸), and 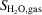, the X-ray scattering calculated for a single gaseous water molecule. Both scattering signals also include Compton scattering.

**Figure 8:**
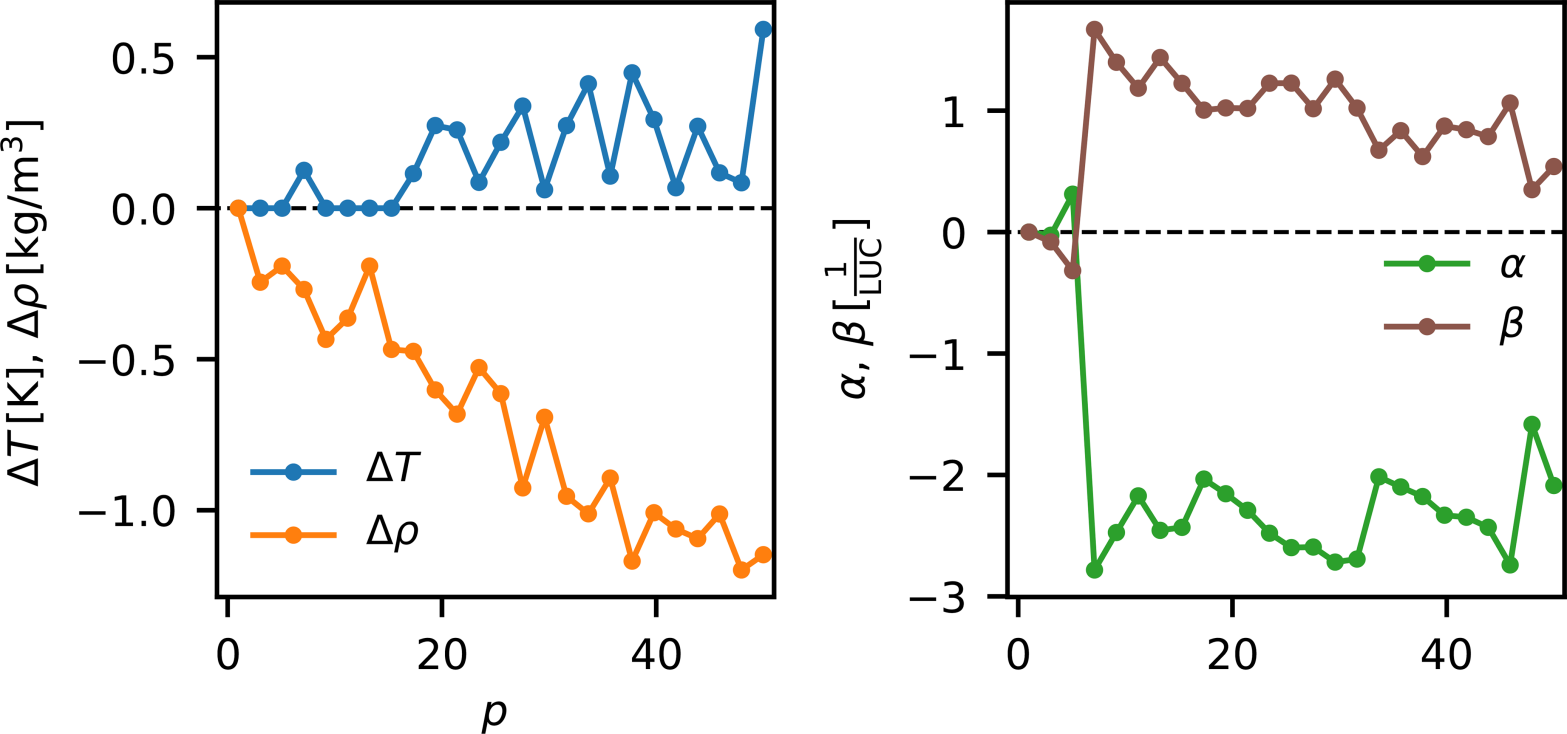
Left: the fitted change in temperature Δ*T* and density Δρ per liquid unit cell (LUC) of the probed volume over the course of the pulse train. Right: the fitted change in the number of probed liquid α and gaseous β solvent molecules per LUC over the course of the train.

To investigate which mechanisms give rise to Δ*S*_PT_, a physical model is employed. The model considers temperature and density changes in the probed region as well as the consequences of severe jet disturbances. Firstly, the highly intense X-ray pulses hitting the sample at kHz repetition rates within the same pulse-train may lead to a local heating of the sample as energy is deposited via absorption of the X-ray pulses. The consequent temperature increase causes the solution to expand on a nanosecond timescale, thus affecting the solvent density. Changes in temperature and density of a given solvent are well known to give rise to characteristic difference scattering signals (Cammarata *et al.*, 2006 ▸; Kjaer *et al.*, 2013 ▸). Such changes in the observed difference scattering signals can be readily modeled using a linear combination of archived difference scattering signals arising from changes in solvent temperature, Δ*S*_Δ*T*_, and density, Δ*S*_Δρ_, for the given choice of solvent, here water (ArchiveLink, 2025 ▸); these contributions are plotted in the left panel of Fig. 7 ▸. Further, as shown by Stan *et al.* (2016 ▸), the intense X-ray pulses may lead to explosions of the liquid jet. From an X-ray scattering point of view, this process causes the scattering contribution from the liquid solvent 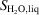 to diminish or, eventually, completely disappear. Although the vaporized solvent may be propelled quite far away from the interaction point, some of it will remain within the interaction volume until the next pulse arrives, contributing to the scattering in gaseous form. Fig. 7 ▸ (right) shows the X-ray scattering signal from liquid water (green curve) and from a gas of water molecules, with the former taken from the work by Skinner *et al.* (2013 ▸) and the latter calculated via the Debye equation for molecular scattering. Compton scattering (Wang *et al.*, 1994 ▸) has been added to both scattering signals. To model the pulse-train difference scattering signals, Δ*S*_PT_, a linear combination fit is constructed from these scattering contributions, analogous to the way Δ*S* is often analyzed (Ihee, 2009 ▸). For each pulse number *p*, a linear combination of the scattering contributions is fitted to Δ*S*_PT_(*q*), 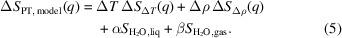
The results of the fit of Δ*S*_PT,model_(*q*, *p*) are presented in the central panel of Fig. 6 ▸. The model reproduces the low *q* features and the negative feature at *q* ≃ 2 Å^−1^ present in Δ*S*_PT_ reasonably well. However, the high *q* features are not well described, suggesting that the model is incomplete. Fig. 8 ▸ shows the pulse dependence of the fitted parameters. The density (orange) decreases over the course of the pulse train to Δρ ≃ −1 kg m^−3^, indicating that the continued energy deposition into the jet from the X-ray and optical laser pulses causes the jet to expand. Δ*T* is very close to zero for the first ∼15 pulses before it increases to ∼0.25 K, with large fluctuations over the rest of the train. The change in the magnitude of the liquid scattering signal, fitted as α, is zero for the first few pulses, until it rapidly decreases with an amplitude corresponding to ∼2 water molecules per liquid unit cell (∼1‰). This drop is concurrent with an increase in scattering from gaseous water, β, of one molecule per LUC, indicating that roughly half of the vaporized water remains within the probed volume.

To summarize the possible series of events: a significant amount of energy from the probe X-ray pulse is absorbed in the jet at the interaction point. This strongly perturbs the jet, increasing the temperature and likely vaporizing the jet around the interaction point in correspondence with what has been previously observed and investigated (Stan *et al.*, 2016 ▸; Hagemann *et al.*, 2021 ▸). This consequent disturbance travels away from the current interaction point as a shock wave, perturbing also the up-stream jet at what will be the next interaction point. Within our modeling framework, this is detected as a decrease in sample density when the upstream jet volume reaches the interaction point. After a few pulses the density decrease is accompanied by a decrease in the amount of sample reaching the interaction point simultaneous with an increase in gas scattering, consistent with the previous observations of jet explosions. After a few more pulses (>6) the energy buildup in the upstream jet is large enough to be detected as a temperature increase of the sample, when the upstream jet material reaches the interaction point.

While the physics-based modeling captures most of the intra-train dynamics shown in Fig. 6 ▸ and provides an explanatory framework for the observations, the rightmost plot of Fig. 6 ▸ shows that a significant residual still remains. This residual is currently believed to stem mainly from structured contributions to acquired data arising from the electronics of the detector system rather than from the X-ray scattering off the sample. In the context of the present work we denote such contributions ‘detector artifacts’ and find them to be multiplicative modulations of the recorded scattering signals as discussed in more detail in Section 4.1 below. First, however, to obtain further insights on the observed intra-train dynamics captured by Δ*S*_PT_, we next turn to model-independent analysis via singular value decomposition.

## 4. Model-independent analysis Δ*S*_PT_

**Figure 9:**
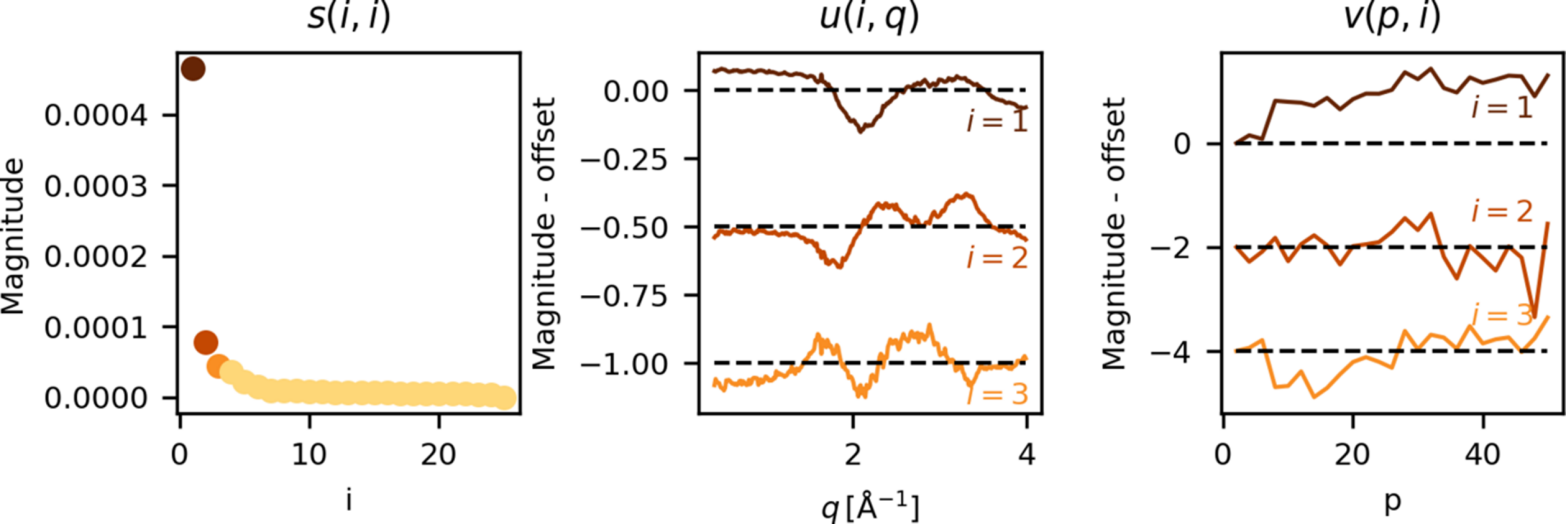
Singular value decomposition of the pulse train difference Δ*S*_PT_(*q*, *p*). Left: *s*(*i*, *i*), the weight of all singular vectors. Middle: *u*(*i* = 1…3, *q*), the first three left singular vectors, denoting the three largest orthogonal contributions to Δ*S*_PT_. Right: *v*(*p*, *i* = 1…3), the first three right singular vectors, describing how *u*(*i*, *q*) depends on the pulse number *p*. These data were measured during experiment A.

Singular value decomposition (SVD) decomposes an *M* × *N* matrix *A* into orthogonal left and right singular vectors *u*(*i*), *v*(*j*) of weight *s*(*i*, *j*), where *i* = 1…*M* and *j* = 1…*N*, *A* = *usv*^*T*^ (Hendler & Shrager, 1994 ▸). In practice, this makes it possible to decompose the pulse train difference, Δ*S*_PT_(*p*, *q*), shown in the left panel of Fig. 6 ▸ into individual orthogonal scattering contributions and their pulse dependence. The weights *s*(*i*, *i*) of all contributions to the SVD are plotted along with the first three (*i* = 1…3) left [*u*(*i*, *q*)] and right [*v*(*p*, *i*)] singular vectors of the SVD of Δ*S*_PT_ in Fig. 9 ▸.

The first right singular vector, [*v*(*p*, 1)], shows a gradual rise with increasing pulse number *p*, indicating that the most dominant feature in the Δ*S*_PT_(*q*,*p*) data matrix intensifies throughout each pulse-train of the X-rays. Such a gradual growth is consistent with the pulse dependence of α and β (Fig. 8 ▸). The second and third left singular vectors [*u*(2, *q*), *u*(3, *q*)] show strong non-zero features of unknown character. Based on the corresponding left singular vectors, [*v*(*p*, 2), *v*(*p*, 3)], these features do not show a clear systematic dependence with pulse number, and the origin of their character requires further analysis. In the following, it is argued that *v*(*p*, 2) likely arises partly from detector artifacts related to the detector memory structure.

### 4.1. Detector artifacts

**Figure 10:**
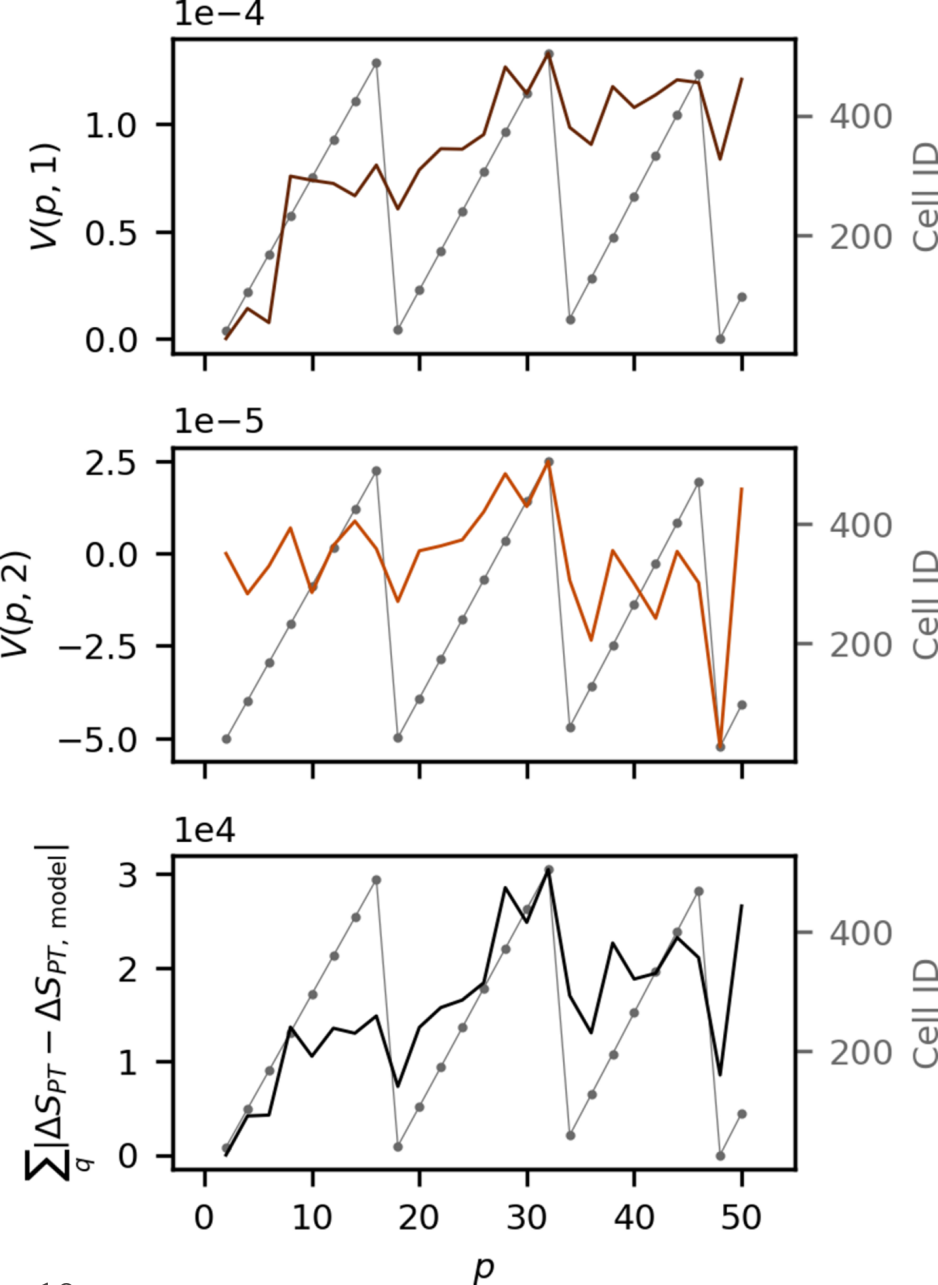
The first (top) and second (middle) right singular vectors of the SVD in Fig. 9 ▸ as well as the residual (bottom) from the model in Section 3 as a function of pulse number *p*. The dashed gray points are plots of the memory cell ID of the memory cell storing scattering from pulse *p*. These data were measured during experiment A.

The LPD is designed to record detector images at the 4.5 MHz base repetition rate of the European XFEL, with the possibility of selecting just a subset of memory cells to store the data, allowing for the possibility of running the experiment at a lower X-ray repetition rate. When operating an experiment at 141 kHz (intra-train) repetition rate, detector images from the 50 consecutive train-pulses are not stored in the 50 consecutive memory cells. Instead, the images are stored in every 32nd cell [as 141 kHz = (1/32)×4.5 MHz] until reaching the last of the 512 memory cells. The storage processing then re-starts from the initial memory cells by filling the empty ones. For example, the first pulses are stored in, for example, memory cell IDs 8, 40, 72, 104, 136, *etc*. Once cell ID 512 is reached, storage re-starts from memory cell ID 9. In practice, this means that for 50 pulses at 141 kHz the detector cycles through the memory cells three times. Fig. 10 ▸ shows the cell ID as a function of pulse number *p* as dashed gray lines plotted alongside the right singular values, *v*(*p*, *i* = 1, 2) from the singular value decomposition of the pulse-train difference matrix Δ*S*_PT_ (top and middle panel). The bottom panel of Fig. 10 ▸ shows a comparison between the memory cell ID and the absolute sum of the residual from the physical model (shown in Fig. 6 ▸, right panel).

**Figure 11:**
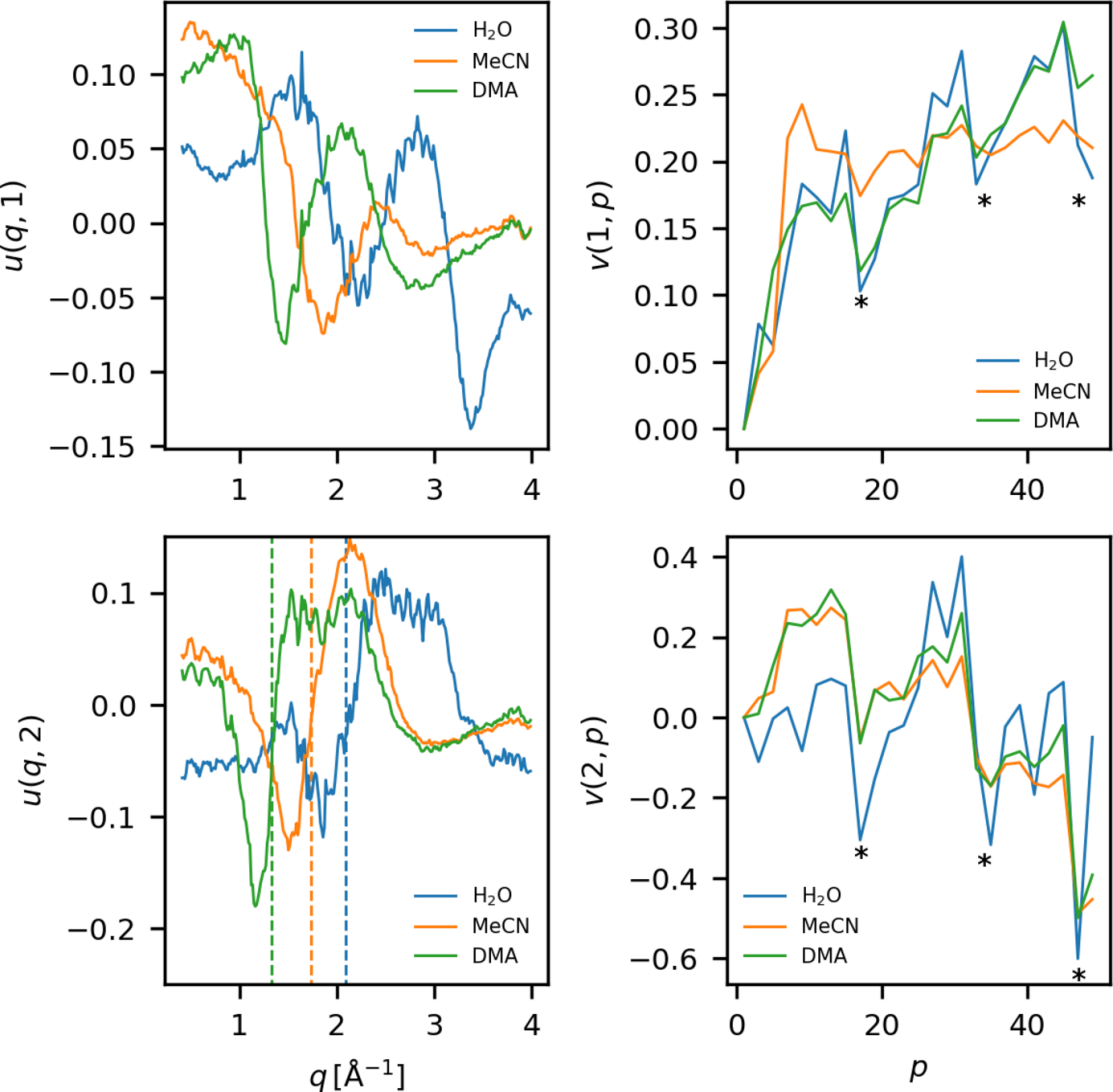
The first two left (left column) and right (right column) singular vectors of the SVD of Δ*S*_PT_ for three different solvents. The dashed lines in the top right panels mark the maximum of the liquid peak for the corresponding solvent. The asterisks (*) in the right column marks where the cycling of the memory cell starts over. These data sets were measured during experiment A.

**Figure 12:**
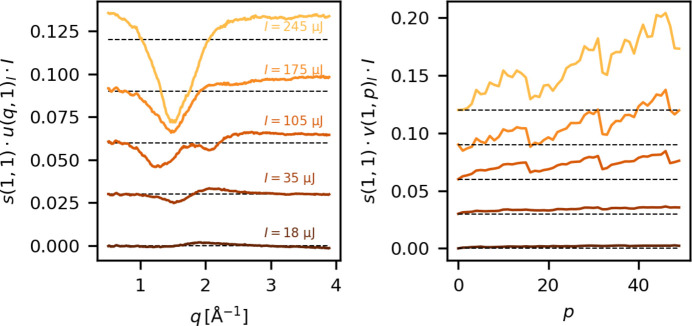
Left: the most dominant left singular vectors, *u*(*q*, *i* = 1), for an SVD of the pulse train difference measured at five different X-ray pulse energies. Right: the corresponding right singular vectors. These data were measured during experiment B.

**Figure 13:**
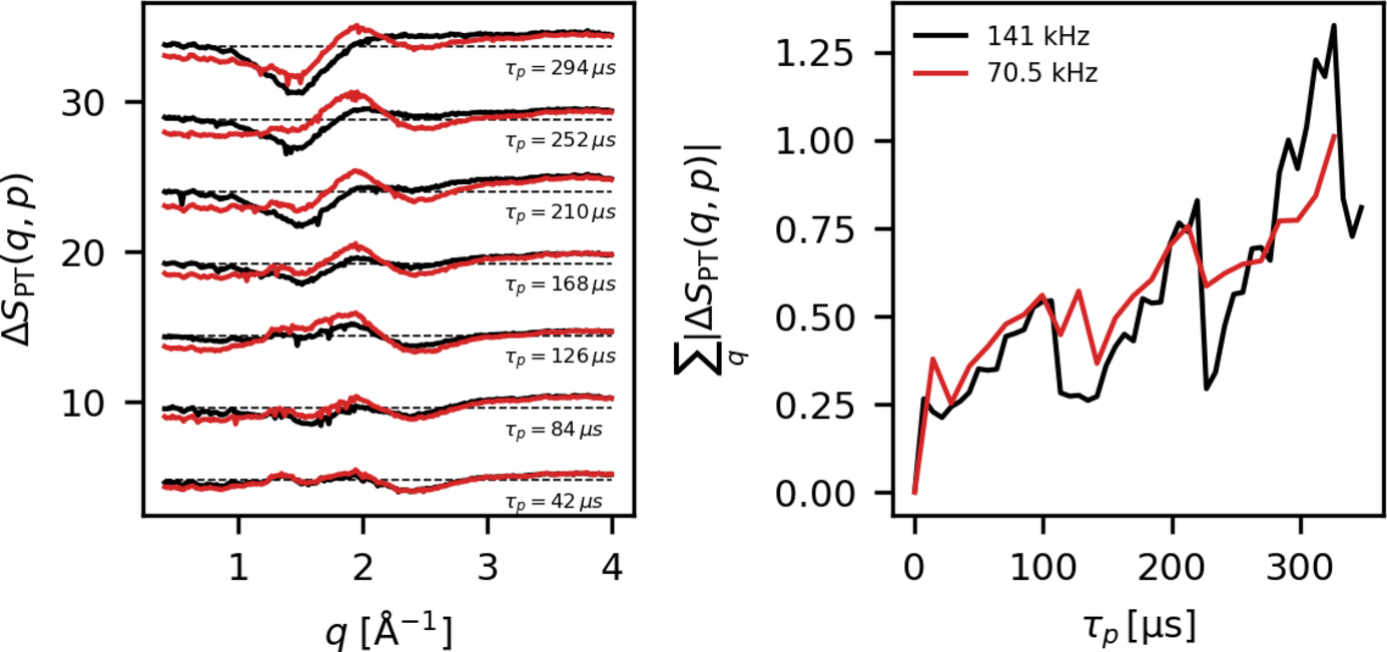
Left: a subset of the pulse train differences Δ*S*_PT_ plotted for a measurement at *f* = 141 kHz (black) and *f* = 70.5 kHz (red) X-ray repetition. Right: the signal magnitude as a function of τ_*p*_ = (*p* − 1)/*f*. Here, the X-ray pulse intensity is ∼35 µJ. These data were measured during experiment B.

From Fig. 10 ▸ some of the abrupt changes in Δ*S*_PT_ (Fig. 6 ▸) can be rationalized, as distinct dips in both the first and second right-singular vectors appear to coincide with the cycling of the memory cells starting over (bottom of the dashed curve), and with a similar pulse evolution in the residual (bottom panel) between the physical model and the acquired pulse-train difference signal. Similar abrupt changes in the acquired data as a function of pulse number can be observed also in Figs. 11–13 below. This correlation between the pulse dependence of Δ*S*_PT_ and the ID of the memory cells which were used to store the scattering data indicates that the behavior stems from the detector. It is assigned to be due to the effect of the so-called memory droop (charge leakage) in the analog storage cells (Koch *et al.*, 2013 ▸) which is a known challenge with the LPD detector, with this charge loss known to be dependent on the operation temperature, the amount of charges stored and the storage time. From these observations, the charge leakage is therefore tentatively concluded to give rise to a multiplicative modulation of the acquired scattering data. This changes along the pulse train, as do the physical changes to the jet, and therefore the model-independent approach based on SVD analysis will not be able to isolate this contribution to the signal. However, further analysis of the residual remaining after subtraction of the physics-based components observed in Δ*S*_PT_, see Fig. 6 ▸ right, may yield insights into how such detector artifacts arising from the basic design of the electronics contribute to TR-XSS experiments. Such an investigation is beyond the scope of the present work which is focused on the direct effects of the high repetition rate of the incident pump- and probe-pulses, and, as demonstrated in Section 3 above, Δ*S*_PT_ arises in part from jet perturbations. The corresponding scattering signature is expected to depend on the hydrodynamic properties of the solvent and, to quantify this further, Δ*S*_PT_ is next compared across different solvents and excitation conditions.

### 4.2. Solvent dependence

Δ*S*_PT_ for the solvents in question, namely water (H_2_O), dimethylacetamide (DMA) and acetonitrile (MeCN), all measured during experiment A with the same X-ray pulse intensity, are plotted in Section S3 of the supporting information. For each of these data sets, an SVD of Δ*S*_PT_ is calculated. Fig. 11 ▸ shows the first two left (left) and right (right) singular vectors of the SVDs. The dashed lines in the bottom left panel mark the maximum position of the liquid peak for each of the solvents.

The first left singular vector, *u*(*q*, 1), is ascribed to the mainly X-ray and laser induced hydrodynamics taking place over the course of the pulse train due to the similarities between its pulse dependence *v*(1, *p*) and the evolution of the components of the physical fit (Fig. 8 ▸). As a result of the different scattering signatures from heating, expansion, *etc*. of the solvents, the scattering contribution, *u*(*q*, 1), is distinctly different for the different solvents. However, *v*(1, *p*) shows a similar pulse dependence for all solvents, a rise, modulated by the effects of memory leakage (described in the previous section).

For all three solvents, *u*(*q*, 2) shows a negative feature followed by a sharp rise. For each of the solvents, the edge of this rise approximately coincides with the maximum of the liquid scattering signal (marked by dashed lines). In other words *u*(*q*, 2) corresponds to a shift of the liquid peak. A comparison between *u*(*q*, 2) and the liquid scattering signal from the corresponding solvent is plotted in Fig. S4. This peak shift could be an effect of a correlation between the X-ray photon energy of a pulse and its position in the train, as a change in X-ray energy is known to give rise to such an effect (van Driel *et al.*, 2015*a* ▸). However, the scattering signal associated with heating and expansion is also associated with a peak shift, making it difficult to completely rule out a hydrodynamic component to *u*(*q*, 2). As mentioned in the discussion of Fig. 10 ▸, *v*(2, *p*) is correlated with memory cells wrapping configuration of this experiment indicating an at least partly electronic origin of the signal. To understand how the effect of these artifacts might be mitigated, an investigation of how Δ*S*_PT_ depends on the X-ray pulse intensity and repetition rate is presented in the following.

## 5. Dependence on X-ray intensity and repetition rate

During experiment B, measurements were performed in the fixed medium gain mode and with systematic variation of the X-ray pulse intensity at two different X-ray repetition rates. This provides an opportunity to investigate how Δ*S*_PT_ depends on these experimental parameters. These measurements were performed in acetonitrile.

The X-ray pulse intensity at 100% beamline transmission is estimated from a downstream gas detector, which provides incident X-ray pulse intensities *I* in units of µJ pulse^−1^, before starting the measurements. Fig. 12 ▸ shows the first singular vectors of the SVD of Δ*S*_PT_ calculated for five scans measured at different incident X-ray pulse intensities, without any laser excitation. All curves have been scaled by the incident X-ray intensity *I*, as this value is proportional to the magnitude of the scattering signal. For the highest intensity, *I* = 245 µJ per pulse, the negative feature at *q* ≃ 1.8 Å^−1^ resembles a decrease in the liquid scattering signal, indicating that at the highest transmissions the deposited energy is enough to partially destroy the liquid jet, vaporizing a significant amount of the solvent and increasing gas scattering (consistent with the results of the modeling discussed in Section 3). As the X-ray transmission is decreased, *u*(*q*, 1) goes from being dominated by a decrease in liquid scattering to being dominated by what appears to be a peak shift for *I* = 35 µJ. This demonstrates that the jet dynamics initiated by the X-rays at high intensity differ significantly from those initiated at low intensity, but that even at the lowest fluence investigated here there are still contributions to the acquired scattering signals arising from beam/sample interactions for the preceding probe pulses.

Fig. 12 ▸ (right) shows the first right singular vectors, *v*(1, *p*), as a function of X-ray intensity. The modulation from the memory cell array wrapping is stronger at higher intensities and seems to disappear almost completely for the lowest intensity. This supports the conclusion that the modulation of Δ*S*_PT_ is caused by charge leakage, as this effect is expected to be proportional to the amount of charge stored in the memory cells. Apart from this, they appear to show roughly the same underlying behavior: a rise over the course of the train. This indicates that a reduction in the X-ray intensity may reduce the prevalence of artifacts in Δ*S*. While the magnitude of Δ*S*_BG_ decreases with decreasing X-ray intensity, so does the magnitude of the difference scattering signal. A comparison between delay scans measured at 185 µJ and 350 µJ is plotted in Fig. S5. Δ*S*_BG_ is observed to be significantly lower for the lower X-ray intensity. This increase in the signal-to-artifact should, however, be weighed against the decreased signal-to-noise.

As a further investigation of how Δ*S*_PT_, and thus Δ*S*_BG_, evolves during the pulse trains, a comparison between two repetition rates is made. For *f*_train_ = 70.5 kHz and 141 kHz, Δ*S*_PT_ is compared based on τ_*p*_, the time elapsed since pulse 1, calculated as τ_*p*_ = (*p* − 1)/*f*, where *f* is the repetition rate in Hz. Fig. 13 ▸ (left) shows a comparison between Δ*S*_PT_(*q*, τ_*p*_) measured at an X-ray repetition rate of 141 kHz (black) and 70.5 kHz (red). The signal shape is initially very similar for the two repetition rates, but diverges after τ_*p*_ ≃ 120 µs. By the end of the train (τ_*p*_ ≃ 300 µs), the pulse-train difference, Δ*S*_PT_, for the higher repetition rate, resembles an overall decrease in the scattering signal, while the lower repetition rate signal mostly resembles a peak shift.

Referring back to the analysis presented in Figs. 6 ▸–8 ▸ above, the differences in sample response seen in Fig. 13 ▸ can be related to the amount of energy absorbed in the sample jet. For the high repetition rate the absorbed energy is enough to partially vaporize the jet, whereas for the lower repetition rate the sample response appears as mostly a change in the hydrodynamic state of the probed sample. Fig. 13 ▸ (right) shows the magnitude of Δ*S*_PT_ for the two different repetition rates, quantified as the absolute sum, 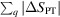, as a function of τ_*p*_. The main difference between the two curves appears to be the magnitude of the detector-related dips discussed above, which are larger for the high repetition rate measurements, as expected from more charge being accumulated. It is interesting that the general magnitude and trend of 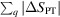 is very similar between the two data sets, even though the number of X-ray pulses interacting with the sample differs by a factor of two and the observed sample response is quite different for the two data sets. For none of the probe beam parameters investigated here could a lower threshold in either intensity of repetition rate be identified, and the contributions from beam/sample dynamics appear already by the second pulse in the train.

## 6. Heuristic approach to correcting Δ*S*

**Figure 14:**
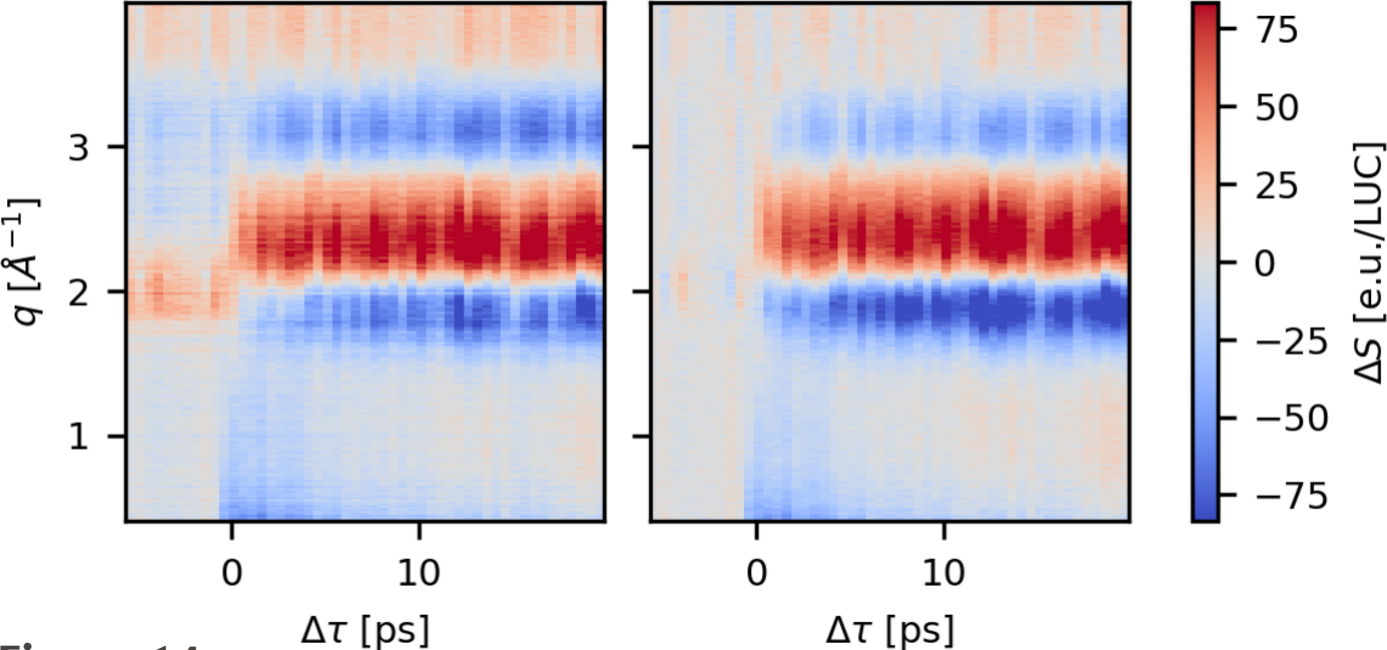
Left: difference scattering constructed from all scattering curves in a scan, according to equation (3); this is the same signal that is plotted in the left panel of Fig. 4 ▸. Right: the same difference scattering signal except that the average of the signal for Δτ < 0 has been subtracted. These data were measured during experiment A.

Artifacts in TR-XSS data usually stem from systematic drifts in X-ray energy, pointing, overlap *etc*. or random shot-to-shot variations as well as detector artifacts (van Driel *et al.*, 2015*a* ▸). For TR-XSS data measured at low repetition rates (order of 100 Hz), as each measurement is independent, artifacts can often be removed by removing scattering curves that are outliers in terms of integrated intensity or correlation between an intensity monitor and integrated intensity (Antolini *et al.*, 2024 ▸). Such characteristics-based filtering does not yield substantial improvement in the signal-to-artifact ratio for data measured at the FXE as the measurements are not independent within each pulse train. Therefore, alternative methods for removing artifacts are needed. Fig. 14 ▸ shows difference scattering signals measured during experiment A. The left panel shows Δ*S* (same as in Fig. 4 ▸) calculated according to equation (3). A clear signal onset is present at Δτ = 0, although for Δτ < 0 a background that appears constant in Δτ is observed. Currently, the best practice to remove this background signal is to subtract a reference signal (Δ*S*_BG_ in Fig. 4 ▸) measured at a negative delay (Lima *et al.*, 2023 ▸), similar to what has also previously been applied for synchrotron TR-XSS studies at MHz repetition rates (Haldrup *et al.*, 2012 ▸). In the present context, this reference signal is determined as the average of Δ*S*(Δτ < −2 ps). The resulting difference between Δ*S* and this reference signal is plotted in the right panel of Fig. 14 ▸. This approach does rather well at removing the artifact(s) observed for Δτ < 0, and thus presumably also removes this contribution to the difference signal for the Δτ > 0 section of the data set.

While the approach of simply subtracting an average of Δ*S*(*q*, τ) for Δτ < 0 from the entire data set performs reasonably well, in the presence of jitter or drifts in X-ray intensity, energy or jet/X-ray overlap, Δ*S*(Δτ < −2 ps) may however not accurately represent the artifact-related contributions for the entirety of a data acquisition sequence lasting perhaps ∼15 min, as for the data shown in Fig. 14 ▸. Indeed, in Fig. 14 ▸ seemingly stochastic variations in Δ*S*(*q*, τ) from time bin to time bin are evident, and, while methods have been developed for subtracting such effects in subsequent analysis (Haldrup, 2014 ▸) or preceding data reduction steps (van Driel *et al.*, 2015*b* ▸), these spurious contributions may be challenging to disentangle from the solute-related sample response of interest, as both can arise from the same thermo- and hydrodynamic phenomena despite differing in their root cause. Such contributions to the acquired data may be mitigated by measuring Δ*S*_BG_ both before and after the actual scan and interpolating between these, or one can employ multiple fast sweeps of the time delays across the scan range as implemented at the XCS instrument at the LCLS. As an alternative to these approaches and directly relevant for the train-structure of the European XFEL, the so-called *alternating* excitation scheme can be employed, as described in the next section.

## 7. Alternating excitation to suppress Δ*S*_Bg_

**Figure 15:**
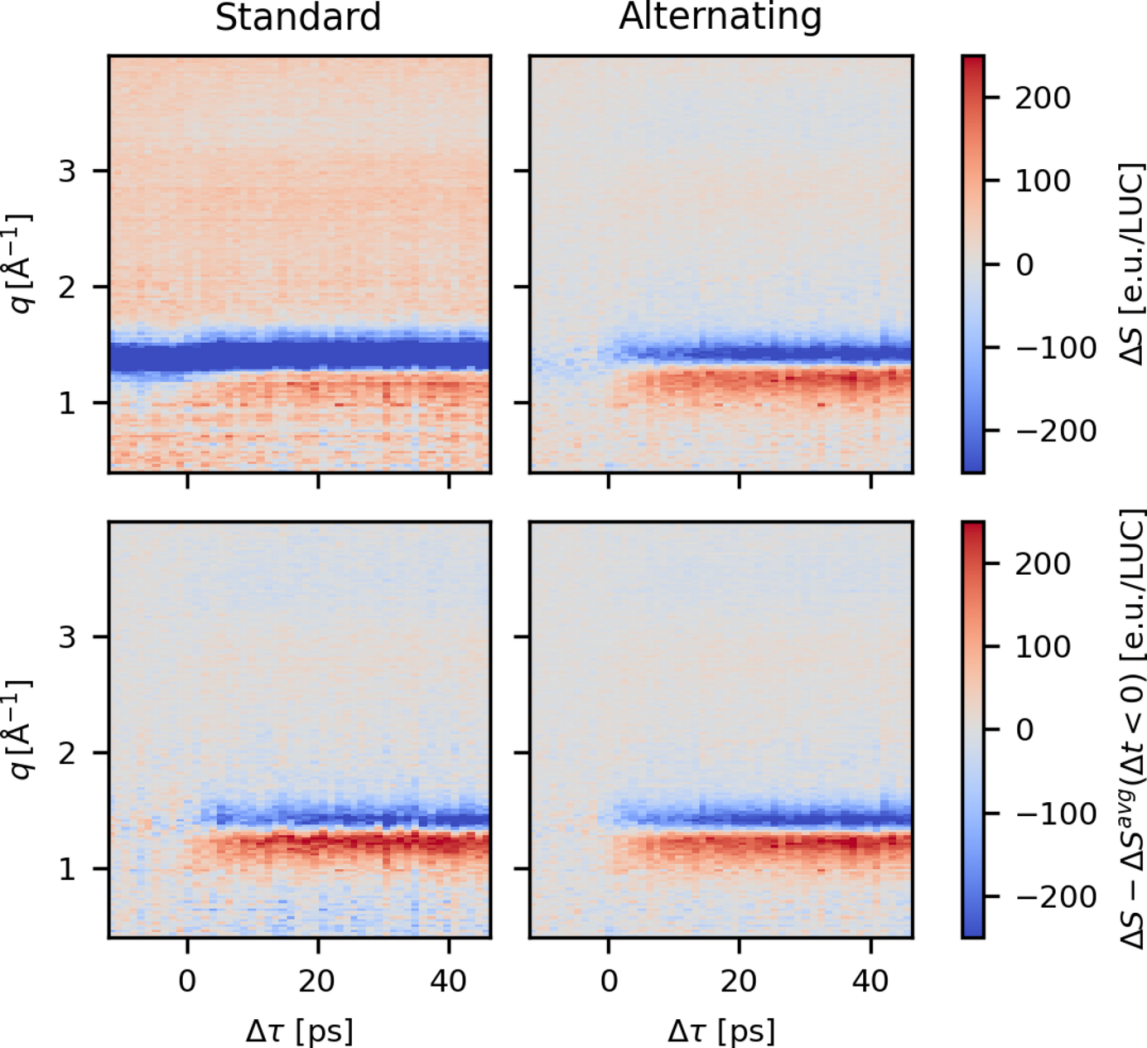
Top row: Δ*S*(*q*, τ) calculated from a measurement using the *standard* and *alternating* excitation patterns, highlighting the much smaller background contribution when the *alternating* scheme is used. Bottom row: the same two difference scattering signals after subtracting a reference signal measured at a negative time delay. These data were measured during experiment C at 30% beamline transmission. The data have been scaled to the LUC using the same approach as described for the water data in Section S1 of the supporting information.

Fig. 2 ▸ above shows a schematic representation of the *alternating* scheme alongside the *standard* excitation scheme. A ‘laser on’ measurement 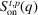 at a pulse number *p* and train number *t* consists of three contributions. The largest contribution is a static contribution, *S*_GS_, from the solute molecules remaining in the ground state and their solvation cages, the bulk solvent, and any static background from air scattering or similar sources. Additionally, 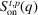 consists of a laser induced contribution, *S*_ES_. Here, *S*_ES_ should be understood as all scattering contributions stemming directly from the electronic excitation of the solute system, *i.e.* structural reorganization of both excited state solute and solvent, solvent heating, as well as a negative contribution from the hole left by photoexcitation in the ground state population (Haldrup *et al.*, 2019 ▸). Lastly, it contains a pulse dependent artifact, 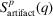, which is what gives rise to the background, Δ*S*_BG_. As outlined in Section 2.1, the next measurement is then a ‘laser off’ measurement 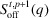, 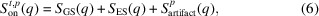

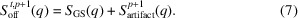
Calculating the nearest neighbor difference and keeping self-normalization implicit to simplify the notation yields
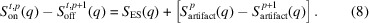
In this expression, it is the second term here that gives rise to the background in Δ*S* when the *standard* excitation scheme is used,
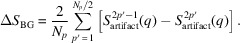
When using the *alternating* excitation scheme, if *S*^*t*,*p*^(*q*) is a ‘laser on’ measurement, the same pulse number *p* in the next train *t* + 1, *S*^*t*+1, *p*^(*q*), will be ‘laser off’. The difference scattering signal, Δ*S*(*q*), can then be constructed as 
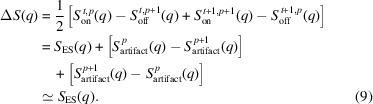
Assuming the artifact contributions to be train-independent due to the long inter-train spacing in time, the contributions from *S*_artifact_ would be expected to cancel out in equation (9) when the difference signal is averaged across a large number of trains. The top row of Fig. 15 ▸ shows a comparison between Δ*S* measured using the *standard* and the *alternating* excitation schemes during experiment C, both with a beamline transmission of 30%. The left panel shows Δ*S*_std_ calculated according to equation (3) and the right panel shows data measured with the *alternating* scheme, exhibiting a much smaller background for Δτ < 0.

While equation (9) suggests that there should be no remaining background contribution evident before τ_0_ when applying the *alternate* excitation scheme, the top-right panel of Fig. 15 ▸ shows that this is not entirely the case. We ascribe this to the presence of jet-related artifacts arising from accumulating perturbations to the liquid jet by the excitation laser. This conclusion is supported by the data shown in Fig. S6, which compares two scans in the *alternating* mode with and without laser excitation to demonstrate how Δ*S*_BG_ is greatly reduced when the excitation laser shutter is closed.

From these observations, applying the *alternating* excitation scheme does not completely eliminate the need to subtract a negative delay reference from Δ*S*. However, using this excitation scheme significantly lowers the magnitude Δ*S*_BG_ as it suppresses the contributions from X-ray induced jet perturbations as well as the those from the systematic differences between the memory cells subsets used for the laser on and laser off scattering patterns. The bottom row of Fig. 15 ▸ shows the final result of this investigation, where 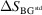 and 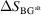 have been subtracted from Δ*S*(*q*, τ)^std^ and Δ*S*(*q*, τ)^alt^. While the two data sets are superficially similar, closer inspection shows a much lower level of variations between adjacent bins in both *q* and τ in these otherwise equi-statistical data sets, *i.e.* a demonstration of significant improvement in the signal-to-artifact ratio when the *alternating* scheme is used.

## 8. Conclusions and outlook

In this work, we presented an analysis of experimental artifacts in time-resolved X-ray solution scattering (TR-XSS) data acquired at high repetition rate XFEL sources, specifically at the FXE instrument at the European XFEL. Using a combination of physics-based modeling and model-independent analysis, we identified and characterized contributions to the difference scattering signal arising from X-ray and laser induced sample perturbations, as well as detector-related artifacts. Our findings indicated that intra-train sample perturbations caused by beam/sample interactions lead to jet heating, expansion and partial vaporization, significantly distorting the measured scattering signals. These effects were particularly pronounced at X-ray fluences exceeding 100 µJ pulse^−1^ and repetition rates above 100 kHz. The resulting perturbations are evident as structured background signals, which may complicate quantitative analysis, especially for low-*Z* solute systems where the signal-to-background ratio is inherently low. We note that in the present investigation with fluences down to 18 µJ pulse^−1^ no threshold for sample perturbations could be identified. Similarly, we observe that lowering the repetition rate from 141 kHz to 70.5 kHz somewhat surprisingly did not change the magnitude of the observed background contributions to the TR-XSS data. However, we note that the sample response was markedly different between the two repetition rates, with no signatures of jet explosions in the probed volume at the lower repetition rate. This result could follow from either the lower amount of deposited energy in the jet or from the signatures of the exploding sample being moved out from the beam interaction region between successive probe events. Finally, in the course of these investigations we found indications that detector artifacts, ascribed to memory cell leakage/charge loss in the LPD detector, introduced small but systematic modulations correlated with the memory cell cycling pattern and pulse number.

To address the data quality issues arising from the high repetition rate, we investigated and compared two background subtraction strategies: one based on identifying and removing signals from regions where the sample response was known *a priori* to be zero, and another based on alternating the laser excitation pattern between probe pulse trains. While both methods proved effective, our results indicated that the alternating excitation scheme offers superior performance, when applicable.

Although the bunch-train time structure of the European XFEL remains unique, we believe that the results presented as well as the analytical approaches developed and demonstrated in this study will prove valuable for establishing best practices in data acquisition and detector characterization for TR-XSS experiments at XFEL facilities. This is in particular pertinent as the general trend for XFEL sources is going in the direction of higher probe pulse intensities and multi-kHz repetition rates, both of which may adversely influence the quality of the data acquired, although the photon counting statistics may of course improve.

## 9. Related literature

The following references, not cited in the main body of the paper, have been cited in the supporting information: Biasin *et al.* (2018 ▸); Chantler *et al.* (2005 ▸).

## Supplementary Material

Supporting Sections S1 to S5, including Figs. S1 to S7. DOI: 10.1107/S1600577525009737/gy5081sup1.pdf

## Figures and Tables

**Table 1:** Experimental details for the three experiments For experiment A, an excitation wavelength of 530 nm was used for the water data, whereas 710 nm was used for the MeCN and DMA data sets.

Proposal No. (label)	Solvent	*E*_X-ray_ (keV)	λ_exc_ (nm)	*E*_exc_ (µJ)	Gain mode
p2787 (A)	Various	9.3	530 or 710	2.8	Auto
p2983 (B)	Acetonitrile	12	490	4.4	Medium
p6736 (C)	thf	9.1	415	1.8	Medium

## References

[bb1] Antolini, C., Sosa Alfaro, V., Reinhard, M., Chatterjee, G., Ribson, R., Sokaras, D., Gee, L., Sato, T., Kramer, P. L., Raj, S. L., Hayes, B., Schleissner, P., Garcia-Esparza, A. T., Lim, J., Babicz, J. T., Follmer, A. H., Nelson, S., Chollet, M., Alonso-Mori, R. & van Driel, T. B. (2024). *Molecules* **29**, 2323. 10.3390/molecules29102323 PMC11124266 38792184

[bb2] ArchiveLink (2025). *Solvent response in TRWAXS*, https://sites.google.com/site/trwaxs/. Accessed: 2025–10–02.

[bb3] Ashiotis, G., Deschildre, A., Nawaz, Z., Wright, J. P., Karkoulis, D., Picca, F. E. & Kieffer, J. (2015). *J. Appl. Cryst.* **48**, 510–519. 10.1107/S1600576715004306 PMC4379438 25844080

[bb4] Biasin, E., Fox, Z. W., Andersen, A., Ledbetter, K., Kjaer, K. S., Alonso-Mori, R., Carlstad, J. M., Chollet, M., Gaynor, J. D., Glownia, J. M., Hong, K., Kroll, T., Lee, J. H., Liekhus-Schmaltz, C., Reinhard, M., Sokaras, D., Zhang, Y., Doumy, G., March, A. M., Southworth, S. H., Mukamel, S., Gaffney, K. J., Schoenlein, R. W., Govind, N., Cordones, A. A. & Khalil, M. (2021). *Nat. Chem.* **13**, 343–349. 10.1038/s41557-020-00629-3 33589787

[bb333] Biasin, E., van Driel, T. B., Levi, G., Laursen, M. G., Dohn, A. O., Moltke, A., Vester, P., Hansen, F. B. K., Kjaer, K. S., Harlang, T., Hartsock, R., Christensen, M., Gaffney, K. J., Henriksen, N. E., Møller, K. B., Haldrup, K. & Nielsen, M. M. (2018). *J Synchrotron Rad*, **25**, 306–315. 10.1107/S1600577517016964 29488907

[bb5] Biednov, M., Yousef, H., Otte, F., Choi, T.-K., Jiang, Y., Frankenberger, P., Knoll, M., Zalden, P., Ramilli, M., Gawelda, W., Canton, S., Lima, F. A., Milne, C. & Khakhulin, D. (2023). *Nucl. Instrum. Methods Phys. Res. A* **1055**, 168540.

[bb6] Bösecke, P. & Diat, O. (1997). *J. Appl. Cryst.* **30**, 867–871.

[bb7] Cammarata, M., Lorenc, M., Kim, T. K., Lee, J. H., Kong, Q. Y., Pontecorvo, E., Lo Russo, M., Schiró, G., Cupane, A., Wulff, M. & Ihee, H. (2006). *J. Chem. Phys.* **124**, 124504. 10.1063/1.2176617 16599694

[bb334] Chantler, C. T., Olsen, K., Dragoset, R. A., Chang, J., Kishore, A. R., Kotochigova, S. A. & Zucker, D. S. (2005). *X-ray Form Factor, Attenuation, and Scattering Tables NIST Standard Reference Database 66.* National Institute of Standards and Technology, Gaithersburg, MD, USA.

[bb8] Choi, E. H., Kim, J. G., Kim, J., Ki, H., Lee, Y., Lee, S., Yoon, K., Kim, J., Kim, J. & Ihee, H. (2021). *Nat. Commun.* **12**, 4732. 10.1038/s41467-021-25070-z PMC8342516 34354075

[bb9] Choi, E. H., Lee, Y., Heo, J. & Ihee, H. (2022). *Chem. Sci.* **13**, 8457–8490. 10.1039/d2sc00502f PMC9337737 35974755

[bb10] Decking, W., Abeghyan, S., Abramian, P., Abramsky, A., Aguirre, A., Albrecht, C., Alou, P., Altarelli, M., Altmann, P., Amyan, K., Anashin, V., Apostolov, E., Appel, K., Auguste, D., Ayvazyan, V., Baark, S., Babies, F., Baboi, N., Bak, P., Balandin, V., Baldinger, R., Baranasic, B., Barbanotti, S., Belikov, O., Belokurov, V., Belova, L., Belyakov, V., Berry, S., Bertucci, M., Beutner, B., Block, A., Blöcher, M., Böckmann, T., Bohm, C., Böhnert, M., Bondar, V., Bondarchuk, E., Bonezzi, M., Borowiec, P., Bösch, C., Bösenberg, U., Bosotti, A., Böspflug, R., Bousonville, M., Boyd, E., Bozhko, Y., Brand, A., Branlard, J., Briechle, S., Brinker, F., Brinker, S., Brinkmann, R., Brockhauser, S., Brovko, O., Brück, H., Brüdgam, A., Butkowski, L., Büttner, T., Calero, J., Castro-Carballo, E., Cattalanotto, G., Charrier, J., Chen, J., Cherepenko, A., Cheskidov, V., Chiodini, M., Chong, A., Choroba, S., Chorowski, M., Churanov, D., Cichalewski, W., Clausen, M., Clement, W., Cloué, C., Cobos, J. A., Coppola, N., Cunis, S., Czuba, K., Czwalinna, M., D’Almagne, B., Dammann, J., Danared, H., de Zubiaurre Wagner, A., Delfs, A., Delfs, T., Dietrich, F., Dietrich, T., Dohlus, M., Dommach, M., Donat, A., Dong, X., Doynikov, N., Dressel, M., Duda, M., Duda, P., Eckoldt, H., Ehsan, W., Eidam, J., Eints, F., Engling, C., Englisch, U., Ermakov, A., Escherich, K., Eschke, J., Saldin, E., Faesing, M., Fallou, A., Felber, M., Fenner, M., Fernandes, B., Fernández, J. M., Feuker, S., Filippakopoulos, K., Floettmann, K., Fogel, V., Fontaine, M., Francés, A., Martin, I. F., Freund, W., Freyermuth, T., Friedland, M., Fröhlich, L., Fusetti, M., Fydrych, J., Gallas, A., García, O., Garcia-Tabares, L., Geloni, G., Gerasimova, N., Gerth, C., Geßler, P., Gharibyan, V., Gloor, M., Głowinkowski, J., Goessel, A., Gołębiewski, Z., Golubeva, N., Grabowski, W., Graeff, W., Grebentsov, A., Grecki, M., Grevsmuehl, T., Gross, M., Grosse-Wortmann, U., Grünert, J., Grunewald, S., Grzegory, P., Feng, G., Guler, H., Gusev, G., Gutierrez, J. L., Hagge, L., Hamberg, M., Hanneken, R., Harms, E., Hartl, I., Hauberg, A., Hauf, S., Hauschildt, J., Hauser, J., Havlicek, J., Hedqvist, A., Heidbrook, N., Hellberg, F., Henning, D., Hensler, O., Hermann, T., Hidvégi, A., Hierholzer, M., Hintz, H., Hoffmann, F., Hoffmann, M., Hoffmann, M., Holler, Y., Hüning, M., Ignatenko, A., Ilchen, M., Iluk, A., Iversen, J., Iversen, J., Izquierdo, M., Jachmann, L., Jardon, N., Jastrow, U., Jensch, K., Jensen, J., Jeżabek, M., Jidda, M., Jin, H., Johansson, N., Jonas, R., Kaabi, W., Kaefer, D., Kammering, R., Kapitza, H., Karabekyan, S., Karstensen, S., Kasprzak, K., Katalev, V., Keese, D., Keil, B., Kholopov, M., Killenberger, M., Kitaev, B., Klimchenko, Y., Klos, R., Knebel, L., Koch, A., Koepke, M., Köhler, S., Köhler, W., Kohlstrunk, N., Konopkova, Z., Konstantinov, A., Kook, W., Koprek, W., Körfer, M., Korth, O., Kosarev, A., Kosiński, K., Kostin, D., Kot, Y., Kotarba, A., Kozak, T., Kozak, V., Kramert, R., Krasilnikov, M., Krasnov, A., Krause, B., Kravchuk, L., Krebs, O., Kretschmer, R., Kreutzkamp, J., Kröplin, O., Krzysik, K., Kube, G., Kuehn, H., Kujala, N., Kulikov, V., Kuzminych, V., La Civita, D., Lacroix, M., Lamb, T., Lancetov, A., Larsson, M., Le Pinvidic, D., Lederer, S., Lensch, T., Lenz, D., Leuschner, A., Levenhagen, F., Li, Y., Liebing, J., Lilje, L., Limberg, T., Lipka, D., List, B., Liu, J., Liu, S., Lorbeer, B., Lorkiewicz, J., Lu, H. H., Ludwig, F., Machau, K., Maciocha, W., Madec, C., Magueur, C., Maiano, C., Maksimova, I., Malcher, K., Maltezopoulos, T., Mamoshkina, E., Manschwetus, B., Marcellini, F., Marinkovic, G., Martinez, T., Martirosyan, H., Maschmann, W., Maslov, M., Matheisen, A., Mavric, U., Meißner, J., Meissner, K., Messerschmidt, M., Meyners, N., Michalski, G., Michelato, P., Mildner, N., Moe, M., Moglia, F., Mohr, C., Mohr, S., Möller, W., Mommerz, M., Monaco, L., Montiel, C., Moretti, M., Morozov, I., Morozov, P., Mross, D., Mueller, J., Müller, C., Müller, J., Müller, K., Munilla, J., Münnich, A., Muratov, V., Napoly, O., Näser, B., Nefedov, N., Neumann, R., Neumann, R., Ngada, N., Noelle, D., Obier, F., Okunev, I., Oliver, J. A., Omet, M., Oppelt, A., Ottmar, A., Oublaid, M., Pagani, C., Paparella, R., Paramonov, V., Peitzmann, C., Penning, J., Perus, A., Peters, F., Petersen, B., Petrov, A., Petrov, I., Pfeiffer, S., Pflüger, J., Philipp, S., Pienaud, Y., Pierini, P., Pivovarov, S., Planas, M., Pławski, E., Pohl, M., Polinski, J., Popov, V., Prat, S., Prenting, J., Priebe, G., Pryschelski, H., Przygoda, K., Pyata, E., Racky, B., Rathjen, A., Ratuschni, W., Regnaud-Campderros, S., Rehlich, K., Reschke, D., Robson, C., Roever, J., Roggli, M., Rothenburg, J., Rusiński, E., Rybaniec, R., Sahling, H., Salmani, M., Samoylova, L., Sanzone, D., Saretzki, F., Sawlanski, O., Schaffran, J., Schlarb, H., Schlösser, M., Schlott, V., Schmidt, C., Schmidt-Foehre, F., Schmitz, M., Schmökel, M., Schnautz, T., Schneidmiller, E., Scholz, M., Schöneburg, B., Schultze, J., Schulz, C., Schwarz, A., Sekutowicz, J., Sellmann, D., Semenov, E., Serkez, S., Sertore, D., Shehzad, N., Shemarykin, P., Shi, L., Sienkiewicz, M., Sikora, D., Sikorski, M., Silenzi, A., Simon, C., Singer, W., Singer, X., Sinn, H., Sinram, K., Skvorodnev, N., Smirnow, P., Sommer, T., Sorokin, A., Stadler, M., Steckel, M., Steffen, B., Steinhau-Kühl, N., Stephan, F., Stodulski, M., Stolper, M., Sulimov, A., Susen, R., Świerblewski, J., Sydlo, C., Syresin, E., Sytchev, V., Szuba, J., Tesch, N., Thie, J., Thiebault, A., Tiedtke, K., Tischhauser, D., Tolkiehn, J., Tomin, S., Tonisch, F., Toral, F., Torbin, I., Trapp, A., Treyer, D., Trowitzsch, G., Trublet, T., Tschentscher, T., Ullrich, F., Vannoni, M., Varela, P., Varghese, G., Vashchenko, G., Vasic, M., Vazquez-Velez, C., Verguet, A., Vilcins-Czvitkovits, S., Villanueva, R., Visentin, B., Viti, M., Vogel, E., Volobuev, E., Wagner, R., Walker, N., Wamsat, T., Weddig, H., Weichert, G., Weise, H., Wenndorf, R., Werner, M., Wichmann, R., Wiebers, C., Wiencek, M., Wilksen, T., Will, I., Winkelmann, L., Winkowski, M., Wittenburg, K., Witzig, A., Wlk, P., Wohlenberg, T., Wojciechowski, M., Wolff-Fabris, F., Wrochna, G., Wrona, K., Yakopov, M., Yang, B., Yang, F., Yurkov, M., Zagorodnov, I., Zalden, P., Zavadtsev, A., Zavadtsev, D., Zhirnov, A., Zhukov, A., Ziemann, V., Zolotov, A., Zolotukhina, N., Zummack, F. & Zybin, D. (2020). *Nat. Photon.* **14**, 391–397.

[bb11] Gaffney, K. (2021). *Chem. Sci.* **12**, 8010–8025. 10.1039/d1sc01864g PMC8208315 34194691

[bb12] Galler, A., Gawelda, W., Biednov, M., Bomer, C., Britz, A., Brockhauser, S., Choi, T.-K., Diez, M., Frankenberger, P., French, M., Görries, D., Hart, M., Hauf, S., Khakhulin, D., Knoll, M., Korsch, T., Kubicek, K., Kuster, M., Lang, P., Alves Lima, F., Otte, F., Schulz, S., Zalden, P. & Bressler, C. (2019). *J. Synchrotron Rad.* **26**, 1432–1447. 10.1107/S1600577519006647 PMC6730617 31490131

[bb13] Hagemann, J., Vassholz, M., Hoeppe, H., Osterhoff, M., Rosselló, J. M., Mettin, R., Seiboth, F., Schropp, A., Möller, J., Hallmann, J., Kim, C., Scholz, M., Boesenberg, U., Schaffer, R., Zozulya, A., Lu, W., Shayduk, R., Madsen, A., Schroer, C. G. & Salditt, T. (2021). *J. Synchrotron Rad.* **28**, 52–63.

[bb14] Haldrup, K. (2014). *Philos. Trans. R. Soc. B* **369**, 20130336. 10.1098/rstb.2013.0336 PMC4052871 24914162

[bb15] Haldrup, K., Christensen, M. & Meedom Nielsen, M. (2010). *Acta Cryst.* A**66**, 261–269. 10.1107/S0108767309054233 20164649

[bb16] Haldrup, K., Levi, G., Biasin, E., Vester, P., Laursen, M. G., Beyer, F., Kjaer, K. S., Brandt van Driel, T., Harlang, T., Dohn, A. O., Hartsock, R. J., Nelson, S., Glownia, J. M., Lemke, H. T., Christensen, M., Gaffney, K. J., Henriksen, N. E., Møller, K. B. & Nielsen, M. M. (2019). *Phys. Rev. Lett.* **122**, 063001. 10.1103/PhysRevLett.122.063001 30822093

[bb17] Haldrup, K., Vankó, G., Gawelda, W., Galler, A., Doumy, G., March, A. M., Kanter, E. P., Bordage, A., Dohn, A., van Driel, T. B., Kjaer, K. S., Lemke, H. T., Canton, S. E., Uhlig, J., Sundström, V., Young, L., Southworth, S. H., Nielsen, M. M. & Bressler, C. (2012). *J. Phys. Chem. A* **116**, 9878–9887. 10.1021/jp306917x 22970732

[bb18] Hendler, R. W. & Shrager, R. I. (1994). *J. Biochem. Bioph. Methods* **28**, 1–33. 10.1016/0165-022x(94)90061-2 8151067

[bb19] Hura, G., Sorenson, J. M., Glaeser, R. M. & Head-Gordon, T. (2000). *J. Chem. Phys.* **113**, 9140–9148.

[bb20] Ihee, H. (2009). *Acc. Chem. Res.* **42**, 356–366. 10.1021/ar800168v 19117426

[bb21] Jay, R. M., Eckert, S., Vaz da Cruz, V., Fondell, M., Mitzner, R. & Föhlisch, A. (2019). *Angew. Chem. Int. Ed.* **58**, 10742–10746. 10.1002/anie.201904761 PMC6771958 31145507

[bb22] Jeong, H., Ki, H., Kim, J. G., Kim, J., Lee, Y. & Ihee, H. (2022). *Bull. Korean Chem. Soc.* **43**, 376–390.

[bb23] Katayama, T., Choi, T. K., Khakhulin, D., Dohn, A. O., Milne, C. J., Vankó, G., Németh, Z., Lima, F. A., Szlachetko, J., Sato, T., Nozawa, S., Adachi, S. I., Yabashi, M., Penfold, T. J., Gawelda, W. & Levi, G. (2023). *Chem. Sci.* **14**, 2572–2584. 10.1039/d2sc06600a PMC9993854 36908966

[bb24] Khakhulin, D., Otte, F., Biednov, M., Bö*mer*, C., Choi, T., Diez, M., Galler, A., Jiang, Y., Kubicek, K., Lima, F. A., Rodriguez-Fernandez, A., Zalden, P., Gawelda, W. & Bressler, C. (2020). *Appl. Sci.* **10**, 995.

[bb25] Ki, H., Choi, S., Kim, J., Choi, E. H., Lee, S., Lee, Y., Yoon, K., Ahn, C. W., Ahn, D. S., Lee, J. H., Park, J., Eom, I., Kim, M., Chun, S. H., Kim, J., Ihee, H. & Kim, J. (2021). *J. Am. Chem. Soc.* **143**, 14261–14273. 10.1021/jacs.1c06088 34455778

[bb26] Kjaer, K. S., Kunnus, K., Harlang, T. C., Van Driel, T. B., Ledbetter, K., Hartsock, R. W., Reinhard, M. E., Koroidov, S., Li, L., Laursen, M. G., Biasin, E., Hansen, F. B., Vester, P., Christensen, M., Haldrup, K., Nielsen, M. M., Chabera, P., Liu, Y., Tatsuno, H., Timm, C., Uhlig, J., Sundstöm, V., Németh, Z., Szemes, D. S., Bajnóczi, É., Vankó, G., Alonso-Mori, R., Glownia, J. M., Nelson, S., Sikorski, M., Sokaras, D., Lemke, H. T., Canton, S. E., Wärnmark, K., Persson, P., Cordones, A. A. & Gaffney, K. J. (2018). *Phys. Chem. Chem. Phys.* **20**, 4238–4249. 10.1039/c7cp07838b 29364300

[bb27] Kjaer, K. S., van Driel, T. B., Harlang, T. C., Kunnus, K., Biasin, E., Ledbetter, K., Hartsock, R. W., Reinhard, M. E., Koroidov, S., Li, L., Laursen, M. G., Hansen, F. B., Vester, P., Christensen, M., Haldrup, K., Nielsen, M. M., Dohn, A. O., Pápai, M. I., Møller, K. B., Chabera, P., Liu, Y., Tatsuno, H., Timm, C., Jarenmark, M., Uhlig, J., Sundstöm, V., Wärnmark, K., Persson, P., Németh, Z., Szemes, D. S., Bajnóczi, É., Vankó, G., Alonso-Mori, R., Glownia, J. M., Nelson, S., Sikorski, M., Sokaras, D., Canton, S. E., Lemke, H. T. & Gaffney, K. J. (2019). *Chem. Sci.* **10**, 5749–5760. 10.1039/c8sc04023k PMC6568243 31293761

[bb28] Kjaer, K. S., van Driel, T. B., Kehres, J., Haldrup, K., Khakhulin, D., Bechgaard, K., Cammarata, M., Wulff, M., Sørensen, T. J. & Nielsen, M. M. (2013). *Phys. Chem. Chem. Phys.* **15**, 15003–15016. 10.1039/c3cp50751c 23918050

[bb29] Koch, A., Kuster, M., Sztuk-Dambietz, J. & Turcato, M. (2013). *J. Phys. Conf. Ser.* **425**, 062013.

[bb30] Kunnus, K., Vacher, M., Harlang, T. C. B., Kjaer, K. S., Haldrup, K., Biasin, E., van Driel, T. B., Pápai, M., Chabera, P., Liu, Y., Tatsuno, H., Timm, C., Källman, E., Delcey, M., Hartsock, R. W., Reinhard, M. E., Koroidov, S., Laursen, M. G., Hansen, F. B., Vester, P., Christensen, M., Sandberg, L., Németh, Z., Szemes, D. S., Bajnóczi, É., Alonso-Mori, R., Glownia, J. M., Nelson, S., Sikorski, M., Sokaras, D., Lemke, H. T., Canton, S. E., Møller, K. B., Nielsen, M. M., Vankó, G., Wärnmark, K., Sundström, V., Persson, P., Lundberg, M., Uhlig, J. & Gaffney, K. J. (2020). *Nat. Commun.* **11**, 634. 10.1038/s41467-020-14468-w PMC6994595 32005815

[bb31] Ledbetter, K., Reinhard, M. E., Kunnus, K., Gallo, A., Britz, A., Biasin, E., Glownia, J. M., Nelson, S., Van Driel, T. B., Weninger, C., Zederkof, D. B., Haldrup, K., Cordones, A. A., Gaffney, K. J., Sokaras, D. & Alonso-Mori, R. (2020). *J. Chem. Phys.* **152**, 074203. 10.1063/1.5139441 32087640

[bb32] Levi, G., Pápai, M., Henriksen, N. E., Dohn, A. O. & Møller, K. B. (2018). *J. Phys. Chem. C* **122**, 7100–7119.

[bb33] Lima, F. A., Otte, F., Vakili, M., Ardana-Lamas, F., Biednov, M., Dall’Antonia, F., Frankenberger, P., Gawelda, W., Gelisio, L., Han, H., Huang, X., Jiang, Y., Kloos, M., Kluyver, T., Knoll, M., Kubicek, K., Bermudez Macias, I. J., Schulz, J., Turkot, O., Uemura, Y., Valerio, J., Wang, H., Yousef, H., Zalden, P., Khakhulin, D., Bressler, C. & Milne, C. (2023). *J. Synchrotron Rad.* **30**, 1168–1182. 10.1107/S1600577523008159 PMC10624029 37860937

[bb34] Markmann, V., Pan, J., Hansen, B. L., Haubro, M. L., Nimmrich, A., Lenzen, P., Levantino, M., Katayama, T., Adachi, S., Gorski-Bilke, S., Temps, F., Dohn, A. O., Møller, K. B., Nielsen, M. M. & Haldrup, K. (2024). *Chem. Sci.* **15**, 11391–11401. 10.1039/d4sc01912a PMC11268492 39055005

[bb35] Merritt, I. C. D., Jacquemin, D. & Vacher, M. (2021). *Phys. Chem. Chem. Phys.* **23**, 19155–19165. 10.1039/d1cp01873f 34195720

[bb36] Mezza, D., Allahgholi, A., Delfs, A., Dinapoli, R., Goettlicher, P., Graafsma, H., Greiffenberg, D., Hirsemann, H., Klyuev, A., Laurus, T., Marras, A., Mozzanica, A., Perova, I., Poehlsen, J., Schmitt, B., Sheviakov, I., Shi, X., Trunk, U., Xia, Q., Zhang, J. & Zimmer, M. (2016). *J. Instrum.* **11**, C11019.

[bb37] Nimmrich, A., Panman, M. R., Berntsson, O., Biasin, E., Niebling, S., Petersson, J., Hoernke, M., Björling, A., Gustavsson, E., van Driel, T. B., Dohn, A. O., Laursen, M., Zederkof, D. B., Tono, K., Katayama, T., Owada, S., Nielsen, M. M., Davidsson, J., Uhlig, J., Hub, J. S., Haldrup, K. & Westenhoff, S. (2023). *J. Am. Chem. Soc.* **145**, 15754–15765. 10.1021/jacs.3c00484 PMC10375522 37163700

[bb38] Polonius, S., González, L. & Mai, S. (2025). *Chem. Sci.* **16**, 11128–11137. 10.1039/d5sc01174d PMC12100520 40417306

[bb39] Skinner, L. B., Huang, C., Schlesinger, D., Pettersson, L. G. M., Nilsson, A. & Benmore, C. J. (2013). *J. Chem. Phys.* **138**, 074506. 10.1063/1.4790861 23445023

[bb40] Stan, C. A., Milathianaki, D., Laksmono, H., Sierra, R. G., McQueen, T. A., Messerschmidt, M., Williams, G. J., Koglin, J. E., Lane, T. J., Hayes, M. J., Guillet, S. A., Liang, M., Aquila, A. L., Willmott, P. R., Robinson, J. S., Gumerlock, K. L., Botha, S., Nass, K., Schlichting, I., Shoeman, R. L., Stone, H. A. & Boutet, S. (2016). *Nat. Phys.* **12**, 966–971.

[bb41] van Driel, T. B., Herrmann, S., Carini, G., Nielsen, M. M. & Lemke, H. T. (2015*b*). *J. Synchrotron Rad.* **22**, 584–591. 10.1107/S1600577515005536 PMC4416674 25931072

[bb42] van Driel, T. B., Kjaer, K. S., Biasin, E., Haldrup, K., Lemke, H. T. & Nielsen, M. M. (2015*a*). *Faraday Discuss.* **177**, 443–465. 10.1039/c4fd00203b 25675434

[bb43] van Driel, T. B., Kjaer, K. S., Hartsock, R. W., Dohn, A. O., Harlang, T., Chollet, M., Christensen, M., Gawelda, W., Henriksen, N. E., Kim, J. G., Haldrup, K., Kim, K. H., Ihee, H., Kim, J., Lemke, H., Sun, Z., Sundström, V., Zhang, W., Zhu, D., Møller, K. B., Nielsen, M. M. & Gaffney, K. J. (2016). *Nat. Commun.* **7**, 13678. 10.1038/ncomms13678 PMC5133712 27892472

[bb44] Veale, M., Adkin, P., Booker, P., Coughlan, J., French, M., Hart, M., Nicholls, T., Schneider, A., Seller, P., Pape, I., Sawhney, K., Carini, G. & Hart, P. (2017). *J. Instrum.* **12**, P12003.

[bb45] Walker, B. J., Musser, A. J., Beljonne, D. & Friend, R. H. (2013). *Nat. Chem.* **5**, 1019–1024. 10.1038/nchem.1801 24256865

[bb46] Wang, J., Tripathi, A. N. & Smith, V. H. (1994). *J. Chem. Phys.* **101**, 4842–4854.

[bb47] Wheater, R. M., Hart, M. D., Veale, M. C., Wilson, M. D., Doblas-Jiménez, D., Turcato, M., Milne, C., Yousef, H. & Khakhulin, D. (2022). *J. Instrum.* **17**, P04013.

[bb48] Wiedbrauk, S., Maerz, B., Samoylova, E., Reiner, A., Trommer, F., Mayer, P., Zinth, W. & Dube, H. (2016). *J. Am. Chem. Soc.* **138**, 12219–12227. 10.1021/jacs.6b05981 27571212

[bb49] Zederkof, D. B., Møller, K. B., Nielsen, M. M., Haldrup, K., González, L. & Mai, S. (2022). *J. Am. Chem. Soc.* **144**, 12861–12873. 10.1021/jacs.2c04505 PMC9305979 35776920

