# Supplementary material for: Analysis strategies for time-resolved X-ray solution scattering at high repetition rate XFEL sources
Source: J Synchrotron Radiat. 2026 Jan 1;33(Pt 1):18–31. doi: 10.1107/S1600577525009737 (PMC12809521; doi:10.1107/S1600577525009737)
Supplement: Supplementary file 1 [file s-33-00018-sup1.pdf]

# Supplementary Information for: "Analysis Strategies for Time-Resolved X-ray Solution Scattering at High Repetition Rate XFEL Sources"

## 1. Heat Deposited by Absorbed single X-ray and laser Pulse

A water jet with a 100 $\mu$m diameter absorbs 6 % of the a 9.5 keV X-ray pulse (Chantler *et al.*, 2005). Assuming an X-ray pulse energy of 0.3 mJ, and that the X-rays are focused to a spot size of radius $r = 10\,\mu$m,

$$\Delta \mathrm{T} = \frac{\Delta E_{abs}}{V_{\mathrm{probed}} \cdot \rho_{\mathrm{H_2O}} \cdot C_{v,\mathrm{H_2O}}} = \frac{[1 - \exp(-\mu_{\mathrm{H_2O}} \cdot d_{\mathrm{jet}})] \cdot E_{\mathrm{X-ray\,pulse}}}{\pi \cdot r^2_{\mathrm{X-ray\,spot}} \cdot d_{\mathrm{jet}} \cdot \rho_{\mathrm{H_2O}} \cdot C_{v,\mathrm{H_2O}}}$$
$$= \frac{0.06 \cdot 0.3 \cdot 10^{-3}\,\mathrm{J}}{\pi (10 \cdot 10^{-4}\,\mathrm{cm})^2 \cdot 100 \cdot 10^{-4}\,\mathrm{cm} \cdot 1\frac{\mathrm{g}}{\mathrm{cm}^3} \cdot 4.186\frac{\mathrm{J}}{\mathrm{gK}}} \approx 140\,\mathrm{K}. \qquad \text{(S1)}$$

Here $\rho$ and $C_v$ are the density and specific heat of water and $V_{probed}$ is the volume of the water probed by the X-ray pulse.

As the absorption cross section $\mu$ depends on the X-ray energy roughly as $E^{-3}_{X-ray}$ for all elements, the absorbed energy and hence the magnitude of the temperature increase depends heavily on which X-ray photon energy is used. As such, conducting a study at higher photon energy can be an efficient way of mitigating the effects of sample jet perturbations assuming that the required $q$-range and available photon flux allows it. Figure S1 shows the estimated temperature increase (from Equation S1, varying $\Delta E_{abs}$ via its dependence on $\mu$) in the probed volume as a function of X-ray

photon energy in the range $E_{X-ray} = 5.5$ - 18 keV, assuming a constant pulse energy of 300 $\mu$J.

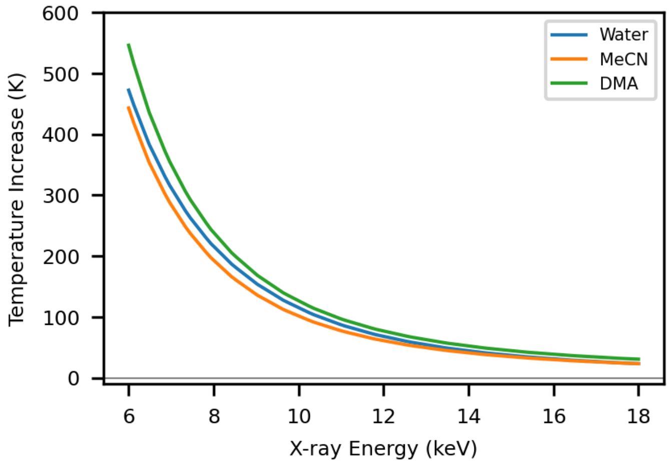


Fig. S1. The temperature increase (equation S1), as a function of X-ray photon energy for the three solvent investigated in this study.

Assuming a typical laser pulse energy of 5 $\mu$J and a sample solution with OD = 1 corresponding to 90 % absorption, the laser-sample interaction results in a temperature increase of roughly 25 % that of the X-rays (assuming the same parameters as in equation S1 and a laser spot with a radius of 30 $\mu$m). Whether this significantly affects the measured signal will depend on the decay pathways for the excited state (vibrational or radiative) as well as on the time scales of these.

## 2. Scaling to the Liquid Unit Cell

To enable quantitative analysis of the difference scattering $\Delta S$ and of the pulse train difference $\Delta S_{PT}$, the data is scaled to the Liquid Unit Cell (LUC). The LUC corresponds to the smallest stochiometrically representative unit. This means that the data is scaled to what would be the scattering, in electron units, from a single solute molecule and counter ion and the number of water molecules in the LUC. This requires

reference scattering signals for the amount of scattering from the solute and counter ions, usually obtained from DFT optimized structures and from the solvent, scaled to a single molecule. In this work the latter is obtained from (Skinner *et al.*, 2013). The left panel of figure S2 shows scattering from the LUC $S_{LUC}$ along with the scaled experimental data. Scattering from the LUC is calculated as,

$$S_{LUC} = S_{[\mathrm{Fe(bpy)(CN)_4}]^{-2}} + 2 \cdot S_{K^+} + 2750 \cdot S_{H_2O}. \tag{S2}$$

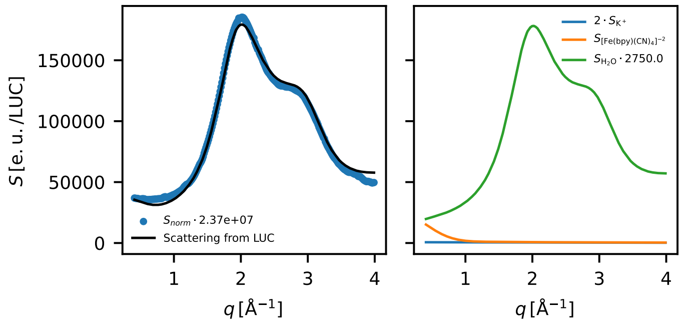


Fig. S2. **Left:** The experimental data scaled to the liquid unit cell (LUC). Scattering from the LUC is described by equation S2. **Right:** The three individual scattering contributions that contribute to the LUC.

The right panel shows the individual scattering contributions that contribute to scattering from the LUC.

## 3. Model Independent Analysis of Solvent Dependence

The pulse train difference, calculated for the three solvents analyzed in the main text are plotted in figure S3. While the X-ray pulse intensity and the number of measurements are the same for all three scans, the excitation conditions are not identical as the water sample was excited at 530 nm, while the MeCN and DMA samples were excited at 710 nm. However, for all three measurements, the excitation wavelength was within the lowest energy band of the visible absorption spectrum of the $[\mathrm{Fe(bpy)(CN)_4}]^{2-}$

sample and power scans were performed to ensure that the data was acquired in a regime where the relationship between excitation power and signal strength was linear.

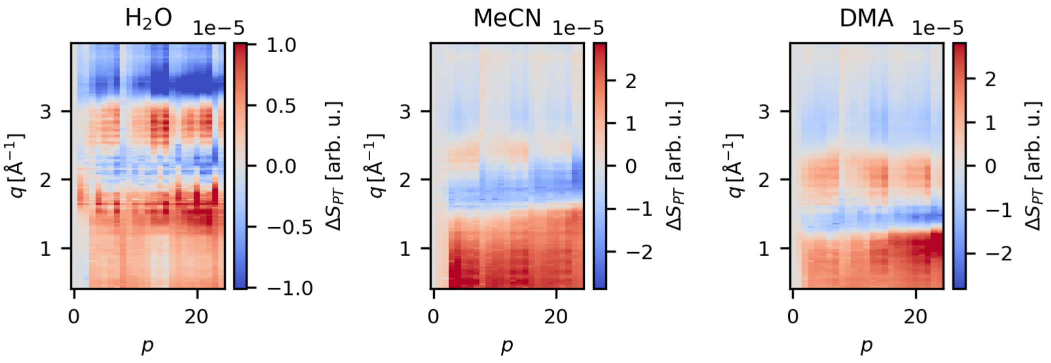


Fig. S3. The pulse train difference, $\Delta S_{PT}$ calculated for scans measured on three different solvents, but with the same X-ray energy.

Figure S4 shows a comparison between the second left singular vector of the SVD of $\Delta S_{PT}$ from the three solvents. The *edge* of $u(q, 2)$ falls right on top of the main peak of the liquid scattering signal for all three samples. As discussed in the main text, this is consistent with a shift of the main liquid peak to higher q.

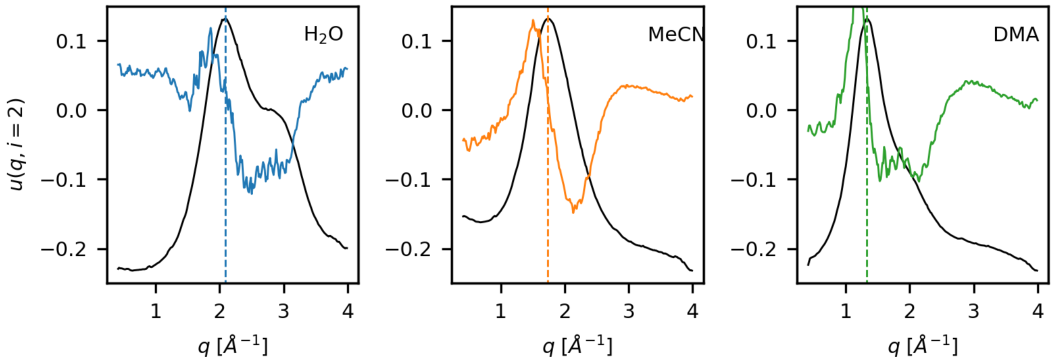


Fig. S4. The second left singular vector of the SVD of $\Delta S_{PT}$ in the three different solvents (colored), plotted on top of the absolute scattering signal. The maximum value of the absolute scattering signal is marked with a dashed line. Note that the rising edge of $u(q, i = 2)$ coincides with the maximum value of the liquid peak.

## 4. Intensity Dependence

Figure S5 shows a comparison between a difference scattering signal measured with a high and one measured with a lower X-ray pulse intensity. The magnitude of $\Delta S_{BG}$ is larger for the data measured at the higher intensity. However, the difference scattering signal measured at the lower intensity fluctuates more (especially at high and low q) as a result of the lower signal-to-noise.

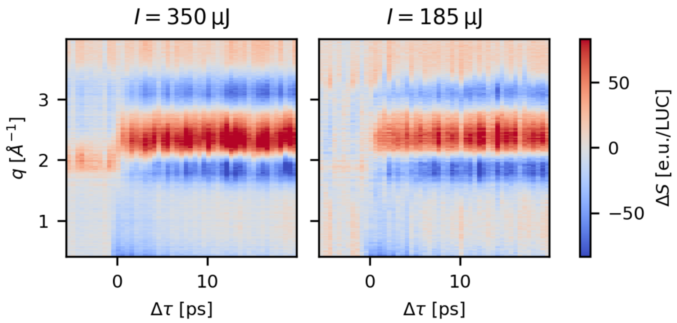


Fig. S5. Comparison between difference scattering signals measured in water during experiment A with two different X-ray pulse intensities.

## 5. Standard vs. Alternating Excitation Scheme

### *5.1. No Laser*

Doing a delay scan with the laser shutter closed makes it possible to remove the effect of the optical laser while the X-ray energy, intensity, repetition rate as well as the detector configuration and all motor positions are identical. Calculating $\Delta S$ according to the standard excitation pattern, $\Delta S$ consists of the same static signal as is observed in the actual delay scan (figure 15 in the main text). When $\Delta S$ is calculated according to the alternating excitation scheme, it is unstructured and appears dominated by statistical noise.

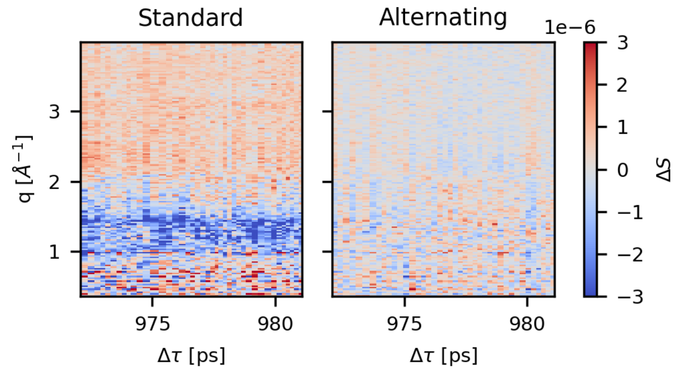


Fig. S6. Comparison of the standard and alternating excitations schemes with the laser shutter closed.

## *5.2. Anisotropic Difference Scattering*

The anisotropic difference scattering signal (Biasin *et al.*, 2018) for a single delay scan ($\sim$ 15 min of data) measured using the standard and the alternating excitation schemes are plotted in figure S7. Using the alternating scheme, only a small contribution from artifacts are present in $\Delta S_2$, leaving mainly the short lived signature of the optical Kerr effect, which has previously been measured and characterized in acetonitrile using TR-XSS (Ki *et al.*, 2021). For the normal excitation scheme, a significant static background is observed before a negative delay reference is subtracted

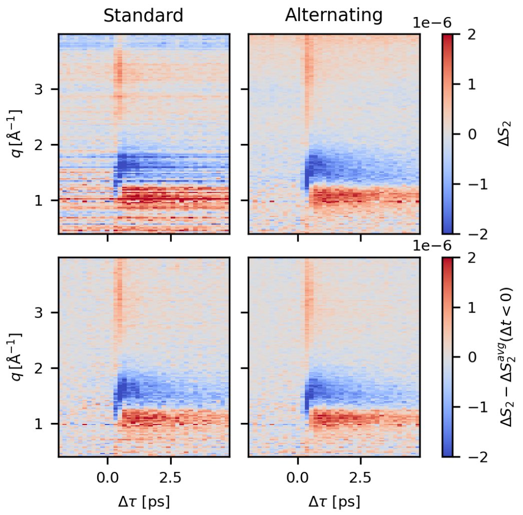


Fig. S7. Comparing the anisotropic scattering signal $\Delta S_2$ measured using the alternating and normal excitation schemes, before and after subtracting a negative time delay reference.

## References


Biasin, E., Van Driel, T. B., Levi, G., Laursen, M. G., Dohn, A. O., Moltke, A., Vester, P., Hansen, F. B., Kjaer, K. S., Harlang, T., Hartsock, R., Christensen, M., Gaffney, K. J., Henriksen, N. E., Møller, K. B., Haldrup, K. & Nielsen, M. M. (2018). *Journal of Synchrotron Radiation*, **25**(2), 306–315.

Chantler, C., Olsen, K., Dragoset, R., Chang, J., Kishore, A., Kotochigova, S. & Zucker, D. (2005). *National Institute of Standards and Technology, Gaithersburg, MD.* .

Ki, H., Choi, S., Kim, J., Choi, E. H., Lee, S., Lee, Y., Yoon, K., Ahn, C. W., Ahn, D. S., Lee, J. H., Park, J., Eom, I., Kim, M., Chun, S. H., Kim, J., Ihee, H. & Kim, J. (2021). *Journal of the American Chemical Society*, **143**(35), 14261–14273.

Skinner, L. B., Huang, C., Schlesinger, D., Pettersson, L. G., Nilsson, A. & Benmore, C. J. (2013). *Journal of Chemical Physics*, **138**(7).
